# Distraction force promotes the osteogenic differentiation of Gli1^+^ cells in facial sutures via primary cilia-mediated Hedgehog signaling pathway

**DOI:** 10.1186/s13287-024-03811-3

**Published:** 2024-07-06

**Authors:** Mengying Jin, Yang An, Zheng Wang, Guanhuier Wang, Zhiyu Lin, Pengbing Ding, Enhang Lu, Zhenmin Zhao, Hongsen Bi

**Affiliations:** 1 Department of Plastic Surgery, Peking University Third Hospital, No. 49 North Garden Road, Haidian District, Beijing, 100191 China; 2 Department of Plastic and Cosmetic Surgery, Henan Provincial People’s Hospital, Henan, China

**Keywords:** Trans-sutural distraction osteogenesis, Gli1^+^ cells, Hedgehog signaling, Primary cilia

## Abstract

**Background:**

Trans-sutural distraction osteogenesis (TSDO) involves the application of distraction force to facial sutures to stimulate osteogenesis. Gli1^+^ cells in the cranial sutures play an important role in bone growth. However, whether Gli1^+^ cells in facial sutures differentiate into bone under distraction force is unknown.

**Methods:**

4-week-old Gli1ER/Td and C57BL/6 mice were used to establish a TSDO model to explore osteogenesis of zygomaticomaxillary sutures. A Gli1^+^ cell lineage tracing model was used to observe the distribution of Gli1^+^ cells and explore the role of Gli1^+^ cells in facial bone remodeling.

**Results:**

Distraction force promoted bone remodeling during TSDO. Fluorescence and two-photon scanning images revealed the distribution of Gli1^+^ cells. Under distraction force, Gli1-lineage cells proliferated significantly and co-localized with Runx2^+^ cells. Hedgehog signaling was upregulated in Gli1^+^ cells. Inhibition of Hedgehog signaling suppresses the proliferation and osteogenesis of Gli1^+^ cells induced by distraction force. Subsequently, the stem cell characteristics of Gli1^+^ cells were identified. Cell-stretching experiments verified that mechanical force promoted the osteogenic differentiation of Gli1^+^ cells through Hh signaling. Furthermore, immunofluorescence staining and RT-qPCR experiments demonstrated that the primary cilia in Gli1^+^ cells exhibit Hedgehog-independent mechanosensitivity, which was required for the osteogenic differentiation induced by mechanical force.

**Conclusions:**

Our study indicates that the primary cilia of Gli1^+^ cells sense mechanical stimuli, mediate Hedgehog signaling activation, and promote the osteogenic differentiation of Gli1^+^ cells in zygomaticomaxillary sutures.

**Supplementary Information:**

The online version contains supplementary material available at 10.1186/s13287-024-03811-3.

## Background

Midfacial hypoplasia (MH) is a clinically common craniofacial skeletal deformity characterized by midfacial depression, narrowing of the upper dental arch, and Class III malocclusion [1, 2]. Treatment options for MH include traditional orthognathic surgery, distraction osteogenesis, and trans-sutural distraction osteogenesis (TSDO) [3–5]. Among these, TSDO involves the application of distraction force to midfacial bones of growing patients, thereby promoting sutural osteogenesis and facial bone remodeling to correct midfacial depression. Compared to other surgeries, TSDO offers advantages such as reduced trauma, avoidance of bone-cutting procedures, lower complication rates, and long-term stability [5, 6]. However, due to the unclear mechanisms underlying TSDO, clinical challenges such as the prolonged distraction period and limited age range for its application persist. Therefore, a comprehensive exploration of the TSDO mechanism is required to optimize current treatment approaches.

The craniofacial skeleton comprises bones such as the nasal bone, maxilla, zygomatic bone, temporal bone, frontal bone, etc. [7, 8]. Fiber connections exist between craniofacial bones, known as craniofacial sutures [9]. These sutures consist of osteogenic fronts (OFs) and the inter-suture mesenchyme [10]. Craniofacial sutures supply the primary sites of osteogenesis with suture mesenchymal stem cells (SuSCs) [1, 11]. In the last decade, research has identified four SuSCs populations within craniofacial sutures postnatally, marked by Axin2, Gli1, Ctsk, and Prrx1, respectively [12–15]. Among these, Gli1^+^ cells within cranial sutures have been demonstrated to possess osteogenic potential and contribute to cranial bone injury repair [14]. However, the distribution and characteristics of Gli1^+^ cells within facial sutures remain unclear. It is yet to be explored whether Gli1^+^ cells in facial sutures differentiate into osteoblasts and facilitate facial bone remodeling under the influence of distraction forces during the TSDO process.

Hedgehog (Hh) signaling is evolutionarily conserved and plays an instructional role in embryonic morphogenesis and organogenesis in various animals [16–18]. It is also considered one of the most important signaling pathways associated with craniofacial development [19]. Currently, three known Hh ligands exist in mammals, including Desert hedgehog (Dhh), Indian hedgehog (Ihh), and Sonic hedgehog (Shh) [20]. Studies have demonstrated that Hedgehog signaling can promote osteogenic differentiation of skeletal stem/progenitor cells [16, 17] and regulate the osteogenic activity of Gli1^+^ SuSCs within cranial sutures during growth and development through the Ihh ligand [14]. Hh signaling plays a crucial role in craniofacial skeletal development, yet whether distraction forces in facial sutures regulate the osteogenic activity of Gli1^+^ cells through Hh signaling remains an unexplored area in the current literature.

Primary cilia are dynamic cellular appendages based on microtubular structures that protrude from the cell surface. They can receive physical and biochemical stimuli and translate them into intracellular signals to regulate growth, development, and tissue homeostasis [21–23]. Hh signaling is one of the most crucial signaling pathways associated with primary cilia [24]. In canonical Hh pathway, when Hh signaling ligands bind to Patched-1 (Ptch1) receptor, it triggers the removal of Ptch1 from the cilia and the enrichment and activation of Smoothened (Smo) within the cilia. This process promotes the generation of Gli transcriptional activators (GliA), further enhancing the transcriptional expression of downstream Hh signaling target genes [25, 26]. As a signaling hub, multiple studies have confirmed that primary cilia possess mechanosensitivity and mediate mechanotransduction in mesenchymal stem cells (MSCs) to promote osteogenesis [27–30]. However, there is currently no research demonstrating the mechanosensitivity of primary cilia in SuSCs and the related mechanotransduction mechanisms.

In this study, we established a Gli1^+^ cell lineage tracing model in C57BL/6 mice, providing the first description of the distribution and cellular characteristics of Gli1^+^ cells within zygomaticomaxillary sutures (ZMS). By applying an anterior-posterior distraction force to the zygomaticomaxillary sutures of mice, we demonstrated that mechanical force could promote the osteogenic activity of Gli1^+^ cells within the suture by activating the Hh signaling pathway, thereby facilitating midfacial growth. Inhibition of Hh signaling suppressed the osteogenesis of Gli1^+^ cells during TSDO and impeded mechanically induced midfacial bone remodeling. In vitro cell stretching experiments validated that mechanical force promoted the osteogenic differentiation of Gli1^+^ cells, confirming that the primary cilia in Gli1^+^ cells possessed mechanosensitivity, mediated Hedgehog signaling activation, and regulated osteogenic differentiation in response to mechanical stimulation.

## Methods

### Animals

Healthy C57BL/6 mice (Animal Department of Peking University Health Science Center, Beijing, China) and Gli1-Cre^ERT2^; R26R^tdTomato^ (Gli1ER/Td) mice (MODEL ORGANISM, Shanghai, China) were used in this study. Ethical clearance was obtained from the Experimental Animal Ethics Committee of Peking University Third Hospital (No. SA2022341). All mice were bred in a pathogen-free environment with a 12-hour light/dark cycle. All the in vivo and in vitro experiments were performed according to the National Institutes of Health regulations for the care and use of animals.

### Drug administration

To induce the activity of Cre, Tamoxifen (Sigma-Aldrich, USA, T5648) was dissolved in corn oil (Sigma-Aldrich, USA, C8267) at 20 mg/ml and injected intraperitoneally (120 mg/kg) daily for 5 days, ending 4 days before surgery or harvesting. GANT61 is a Gli transcription factor antagonist. To inhibit Hh signaling pathway in vivo, GANT61 (MedChemExpress, USA, HY-13,901) was dissolved according to the manufacturer’s instructions and was intraperitoneally injected (40 mg/kg) every other day from 3 days before surgery until the mice were harvested. To inhibit Hh signaling pathway in vitro, GANT61 (10 µM) was added into the culture medium.

### Surgery

For inclusion/exclusion criteria, 4-week-old Gli1ER/Td and C57BL/6 mice with normal and similar weight (12–16 g), appearance, and hair were included. Mice with dislocated distraction devices were excluded. Single-blinding method was used in our study. Mice were randomly assigned to each group using a random number table. The individual mouse was considered the experimental unit, and the method of resource equation approach was used to determine the sample size. Eighty-one C57BL/6 mice and fifty-four Gli1ER/Td mice were included and assigned to 3 groups separately: sham operation group (Control), stretch group (Stretch), and GANT61 + stretch group (G-Stretch). There were no exclusions in this study. The titanium nickel alloy wire with a diameter of 0.25 mm was used to make an elastic W-type distraction device (Beijing NiTi Nuo Technology Co., Ltd, China), which provided a stable distraction force of about 30 g when compressed to 7 mm. Mice were intraperitoneally injected with 0.3% pentobarbital sodium (150 µl/10 g) anesthetic. The skin about 1 mm from the infraorbital edge was cut and the muscle fascia tissue around the zygomatic arch was stripped to expose the bone. The posterior end of the zygomatic arch was drilled with a 0.25 mm diameter hole. The anterior end of the W-type distraction device was placed into the inner edge of the zygomatic arch, and the posterior end was placed into the hole. For Control group, the W-type distraction device was not placed, and other operations were the same. The animal’s temperature was maintained during surgery by using a heating pad. After modeling, animals in each group were sacrificed by cervical dislocation 3, 7, and 14 days after surgery and the skulls were harvested for subsequent experiments.

### Hematoxylin eosin (HE) and Masson staining

Zygomatic arches were isolated from C57BL/6 mice and fixed in 4% paraformaldehyde (Solarbio, China) overnight at room temperature (RT), followed by decalcification in 0.5 M EDTA (Servicebio, China, G1105) for 14 days. Decalcified samples were embedded in paraffin and then sectioned at 5 μm thickness along the sagittal plane. HE staining and Masson staining were performed according to standard procedure.

### Micro-computed tomography (micro-CT) imaging and analysis

Before scanning, the skulls of C57BL/6 mice were fixed and stored in 4% paraformaldehyde. Samples were scanned using a Micro-CT machine (PerkinElmer, USA, Quantum FX). The bone structural parameters, including bone volume (BV) and bone mineral density (BMD), were analyzed using the system in the Micro-CT machine. Mimics 20.0 (Materialise, Belgium) was used for three-dimensional (3D) reconstruction and morphological measurement. The length of the zygomatic arch was defined as the distance between the most anterior point of zygomatic process and the most posterior point of the zygomatic bone.

### Two-photon laser scanning image acquisition

To observe the distribution of Gli1^+^ cells and trace the lineage cells, Gli1ER/Td mice were induced by tamoxifen and then the zygomatic arch was fixed in 4% paraformaldehyde overnight and stored in phosphate buffered saline (PBS) at 4℃ avoiding light before images acquired by a two-photon laser scanning microscope (TCS-SP8 DIVE, Leica, Germany). The excitation beam was focused into the sample plane using a 40x objective lens (NA = 0.75). Fluorescence emission was then collected by photomultiplier tubes with proper dichroic and filter settings corresponding to fluorophores of interest. A laser pulsing at 1045 nm was used for acquiring second harmonic generation (SHG) signal of collagen and fluorescence of tdTomato, which was detected using 560–643 nm band pass filters. All images were acquired and processed in LAS X software (Leica, Germany). Briefly, the X, Y, and Z ranges were adjusted in the acquisition mode to cover complete sutures and adjacent bones. All image stacks were acquired at 8 μm intervals and were used to reconstruct 3D models and obtain optical sectioning.

### Reverse transcription–quantitative PCR (RT-qPCR)

Intact zygomaxillary sutures and adjacent bones of about 0.5 mm were used to extract total RNA using TRIzol reagent (ThermoFisher Scientific, USA). Bilateral tissues of one C57BL/6 mice contributed to one sample collection. Similarly, Gli1^+^ cells were scraped and collected with TRIzol reagent. RNA was reverse-transcribed to cDNA and 2 X qPCR Master Mix (Tiangen, China) was used for RT-qPCR (for primers, see Table S1). ΔΔCt method was used for calculating expression.

### Immunofluorescence (IF) staining

Zygomatic arches were isolated from Gli1ER/Td mice and fixed in 4% paraformaldehyde overnight at 4℃ avoiding light, followed by decalcification in 0.5 M EDTA for 14 days. Decalcified samples were embedded in O.C.T. compound (SAKURA, Japan) and then sectioned at 8 μm thickness along the sagittal plane to perform Runx2 and Ihh staining. Briefly, sections were permeabilized at room temperature for 15 min using 0.2% TritonX-100 (Beyotime, China). Sections were blocked using 3% BSA (Beyotime, China) at 37℃ for 30 min and were then incubated with primary antibodies at 4℃ overnight. On the following day, the samples were incubated with the respective secondary antibodies in the dark for 1 h at 37℃; and nuclei were dyed using DAPI for 10 min.

Primary cilia staining was conducted in Gli1^+^ cells. The cells were fixed with 4% paraformaldehyde for 15 min and then incubated for 5 min using 0.2% TritonX-100. The subsequent operation was consistent with the section staining. Images were captured by Leica TCS-SP8 DIVE and Zeiss confocal microscope. The following antibodies were used in our study: Runx2 (1:200; CST, USA, 12,556 S), Ihh (1:200; Proteintech, USA, 13388-1-AP), anti-acetylated α-tubulin (1:1000, Sigma-Aldrich, USA, T7451), Goat Anti-Mouse IgG (1:200; Abcam, UN, ab150115), and Anti-rabbit IgG (1:200; CST, USA, 4412 S).

### SuSCs isolation and culture

Intact zygomaxillary sutures in 6 tamoxifen-induced 4-week-old Gli1ER/Td mice were excised along with about 0.5 mm of adjacent bone and minced into small pieces. The tissue blocks were evenly spread into a 6 cm dish (Corning, USA) and incubated with high glucose DMEM (HyClone, USA) + 20% FBS (Invitrogen 10437-085) at 37 °C. After 6 h, 3 ml complete medium was added to the dish. Upon reaching 80% confluency, primary cells were digested with 0.25% trypsin and sub-cultivated. Passage 4–6 cells were used for the following experiments.

### Fluorescence-activated cell sorting (FACS) and flow cytometry (FCM) analysis

To isolate Gli1^+^ populations, SuSCs were subjected to cell sorting according to the fluorescence intensity of tdTomato using FACS Aria-II (BD Biosciences, USA). OriCell Mice MSC analysis kit (Oricell, China, MUXMX-09011) was employed to detect the surface markers of SuSCs and Gli1^+^ cells. Cells were digested into single cells with 0.25% trypsin and resuspended with FCM buffer (PBS containing 0.1% BSA). The density of cells was adjusted to 3 × 10^6^ cells/ml. 100 µl of cell suspension was transferred into EP tubes. Primary antibodies were added to each tube (1:50) separately and incubated with the cells for 30 min at 4℃. Then, the cells were washed twice and resuspended with 100 µl buffer, incubated with respective secondary antibodies (1:50) for 30 min at 4℃. After being washed twice, the labeled cells were resuspended with 300 µl buffer and analyzed by FCM (BD Biosciences, USA, Accuri C6).

### Clonal culture and multipotential differentiation

For clonal culture, 1000 Gli1^+^ cells were plated in a 10 cm dish (Corning, USA) and incubated with 10 ml culture medium at 37℃ under hypoxic conditions (5% O2, 5% CO2, balanced with Nitrogen). Clones were observed 7 days after plating. For multipotential differentiation, Gli1^+^ cells were plated in a 6-well plate and reached 80–100% confluency. Osteogenic Differentiation Kit (HyCite, China, BMMB-D101), Adipogenic Differentiation Kit (HyCite, China, BMMB-D102), and Chondrogenic Differentiation Kit (HyCite, China, BMMB-D203) were employed to detect the multi-potent differentiation capability of Gli1^+^ cells according to the standard protocol, respectively. After 21 days of induction, the osteogenic, adipogenic, and chondrogenic capability was assessed using alizarin red staining, oil red O staining, and Alcian blue staining respectively.

### Small interfering (si)RNA transfection

Gli1^+^ cells were transfected with 50 nM siRNA targeting IFT88 or with a scrambled sequence (negative control siRNA) for 48 h using riboFECT CP Transfection Kit (RiboBio, China, C10511-05). The siRNA mentioned above was synthesized by RiboBio Co., Ltd. (Guangzhou, China). IFT88 siRNA transfection efficiency was assessed by RT-qPCR and immunofluorescence staining of primary cilia.

### Stretching of Gli1^+^ cells

Gli1^+^ cells were seeded into cell stretching chambers (4 cm*4 cm; Dongdi Beijing Technology Co., Ltd, China) at a density of 2*10^5^ per chamber. After adherence, the chambers were placed into the stretching machine CELL TANK (Dongdi Beijing Technology Co., Ltd, China). The stretching parameters were set as 0.5 Hz sinusoidal wave and 8% elongation for 6 h each day. The chambers of Control group were placed into the same incubator without any stretching. After 3 days, cells of each group were performed RT-qPCR and IF. Each experiment was repeated three times.

### Statistical analysis

GraphPad Prism 9.4.1 was used for statistical analysis. All statistical data were expressed as mean (± SD). Comparison between groups was statistically analyzed by two-tailed unpaired Student’s t-test. Each group contains at least three independent biological replicates. P-values less than 0.05 were considered statistically significant. *P < 0.05, **P < 0.01, ***P < 0.001, ****P < 0.0001.

## Results

### Distraction force stimulates the osteogenesis in the zygomaticomaxillary suture

**Fig. 1:**
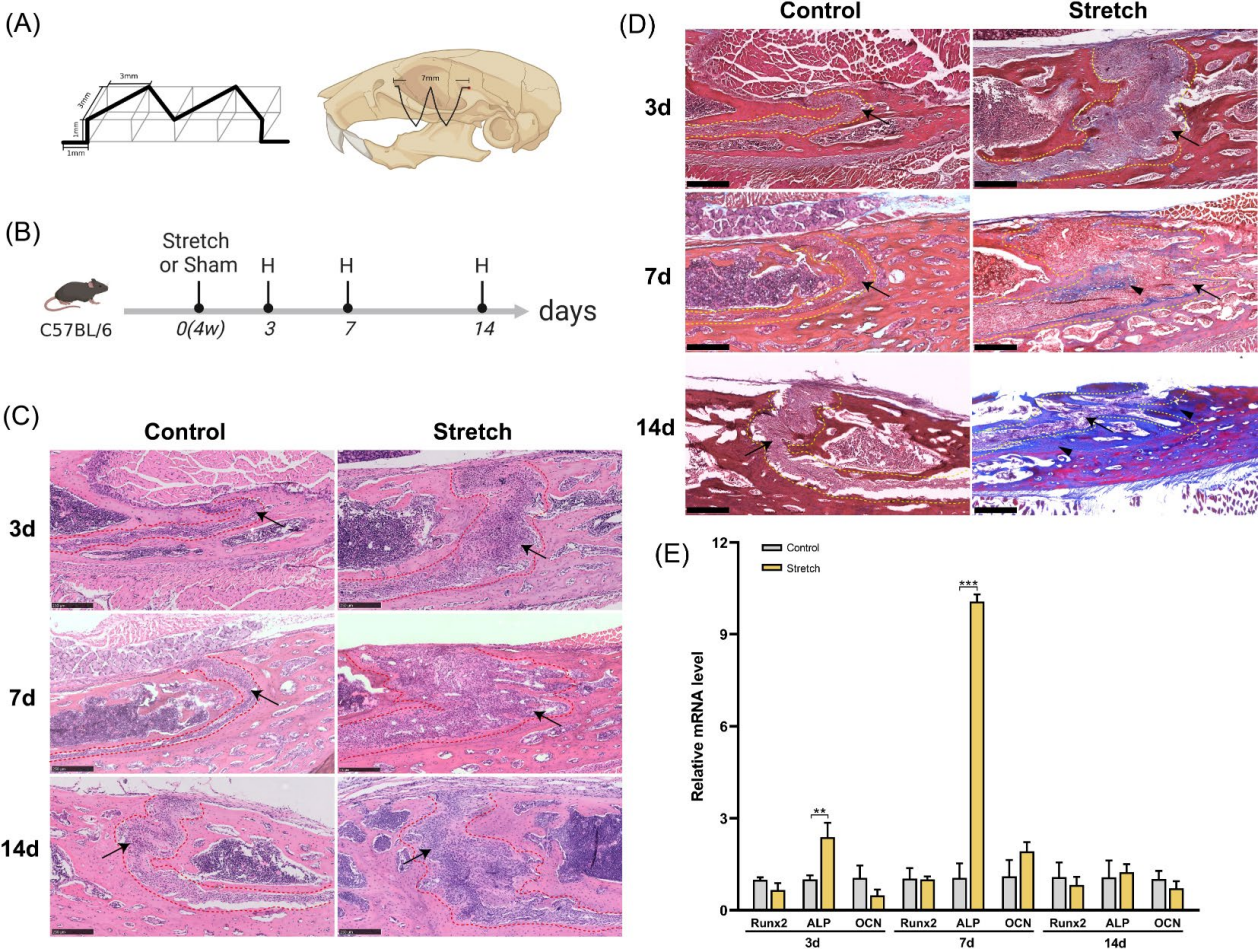
Cytohistological changes of zygomaticomaxillary suture (ZMS) under distraction force. (**A**) Schematic diagram of W-type distraction device and surgical drilling position. (**B**) Experimental design: C57BL/6 mice were harvested after 3, 7, and 14 days of stretching. The sham-operated mice were used as control. H: harvested. (**C**) Histological changes of ZMS after stretching for 3, 7, and 14 days (HE staining). Red dotted line: osteogenic fronts (OFs). Scale bar: 250 μm. Arrows: ZMS. (**D**) Histological changes of ZMS after stretching for 3, 7, and 14 days (Masson staining). Yellow dotted line: OFs. Scale bar: 250 μm. Arrows: ZMS. Arrowheads: new bone. (**E**) RT-qPCR analysis of osteogenesis-related genes of ZMS at 3, 7, and 14 days after surgery. **P < 0.01, ***P < 0.001

To investigate osteogenesis induced by mechanical stimulation, stretching surgery was performed on 4-week-old mice to provide a sagittal distraction force in the ZMS (Fig. 1A), and the tissues were harvested 3, 7, and 14 days after surgery (Fig. 1B). The results of HE staining demonstrated that the sutures were considerably widened, and the cells proliferated without obvious inflammatory infiltration after stretching for 3 and 7 days (Fig. 1C). On day 3, small protuberances were observed at the margins of the sutures without obvious new bone formation. On day 7, vascular-like structures appeared in the sutures, and the OFs became interlaced with the formation of finger-like bone. After stretching for 14 days, hump-like bone appeared in the OFs with a partially restored suture width. The cells in the suture were elongated along the force direction, and the boundary between the suture and edges of the bone became blurred. The sutures in the Control group became slightly wider from day 3 to 14 and the cells in the suture were arranged regularly with relatively smooth OFs (Fig. 1C). Consistent with the results of HE staining, Masson staining showed finger-like new bone formation 7 days after stretching, and massive new bone formation promoted the restoration of sutural width 14 days after stretching (Fig. 1D). Furthermore, we analyzed the expression levels of osteogenesis-related genes in the ZMS (Fig. 1E). Although the expression of Runx2 and OCN did not change significantly, ALP expression was significantly elevated after stretching for 3 and 7 days. After 14 days of stretching, there was no significant difference in the expression of Runx2, ALP, or OCN compared to that in Control.

**Fig. 2:**
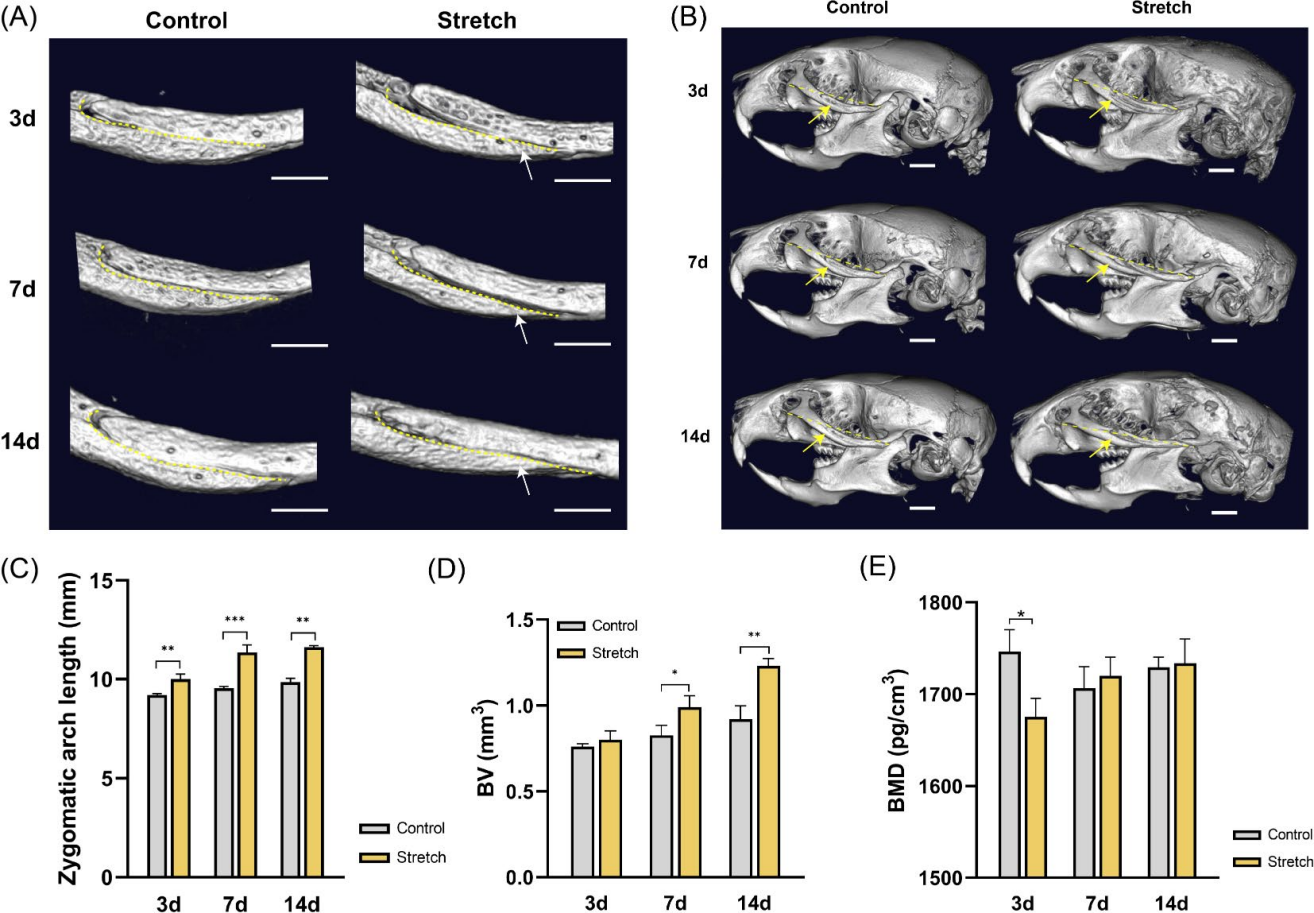
Micro-CT scanning and analysis of the skull after 3, 7, and 14 days of surgery. (**A**) Morphological changes of zygomaticomaxillary suture (ZMS) after stretching. Scale bar: 1 mm. Yellow dotted line: ZMS. Arrows: the curvature of the ZMS decreased during stretching. (**B**) Morphological changes of zygomatic arch and skull after stretching. Yellow dotted line: length of zygomatic arch. Scale bar: 1 mm. Arrows: zygomatic arch. (**C**–**E**) Changes in the length of the zygomatic arch and bone structural parameters of the bone around ZMS. BV: bone volume. BMD: bone mineral density. *P < 0.05, **P < 0.01, ***P < 0.001

Micro-CT scanning and 3D model reconstruction of the skulls were performed to measure the morphological changes and analyze the bone structure parameters of the bone around the ZMS. The results showed that the curvature of the ZMS decreased gradually during the 14 days of stretching, and as the ZMS elongated along the sagittal direction (Fig. 2A). The lengths of the zygomatic arch and head gradually increased (Fig. 2B). Quantitative analysis results showed that the zygomatic arch length in the Stretch group increased compared to that in the Control group (Fig. 2C) at 3, 7, and 14 days after surgery. The measurement results of bone-related parameters showed that there was no statistical difference in the BV around the ZMS between the Stretch and Control groups (Fig. 2D) at the early stage of stretching (3 days). 7 and 14 days after surgery, the BV around the ZMS in the Stretch group was significantly higher than that in the Control group (Fig. 2D). 3 days after surgery, the bone density in the Stretch group was significantly lower than that in the Control group (Fig. 2E). After stretching for 7 and 14 days, there was no significant difference in the BMD between the two groups (Fig. 2E).

Collectively, these data demonstrate that distraction force induces osteogenesis in the ZMS and promotes midfacial growth in mice in the sagittal direction.

### The distribution of Gli1^+^ cells in zygomaticomaxillary sutures of growing mice

**Fig. 3:**
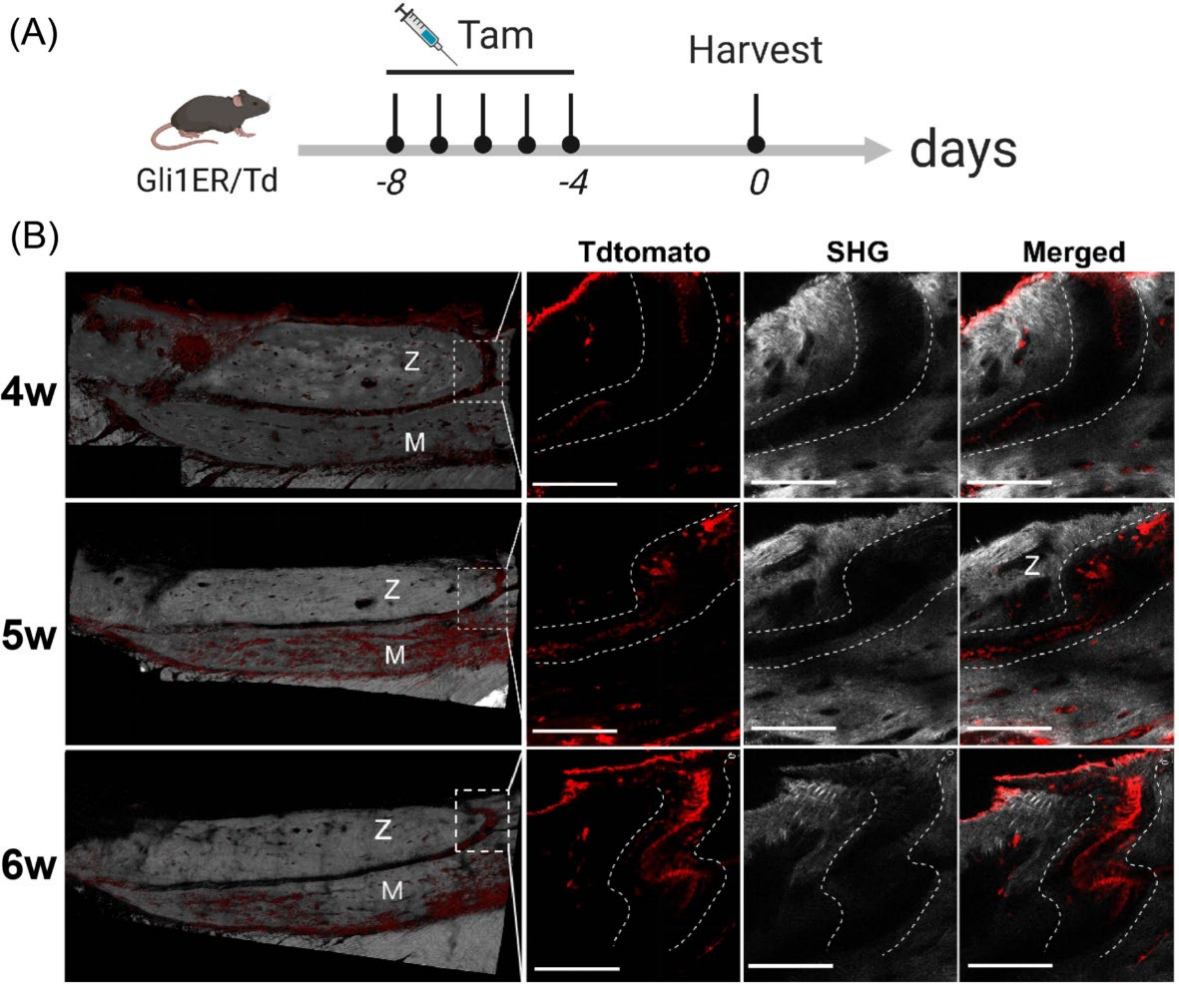
The distribution of Gli1^+^ cells in zygomatic and zygomaticomaxillary sutures (ZMS) of growing mice. (**A**) Gli1ER/Td mice aged 4, 5, and 6 weeks were induced with tamoxifen for 5 days. Tam: tamoxifen. (**B**) Two-photon laser scanning was used to describe the distribution of Gli1^+^ cells. The three-dimensional reconstructed models demonstrate that Gli1^+^ cells were mainly distributed in the suture mesenchyme, bone marrow cavity, and periosteum. Regions in boxes are magnified in the right panel to display the details of the distribution of Gli1^+^ cells in ZMS by optical sections. Z: zygoma. M: maxilla. Red: Gli1^+^ cells (Tdtomato). Gray: second harmonic signal (SHG) of bone collagen. Curved dotted line: osteogenic fronts (OFs). Scale bar: 200 μm

4/5/6-week-old Gli1ER/Td mice were treated with tamoxifen to induce the activity of fluorescent protein tdTomato to characterize the distribution of Gli1^+^ cells in zygomaticomaxillary sutures (Fig. 3A). Images, including the SHG and tdTomato fluorescence signals were obtained by two-photon laser scanning. The bone was identified by the SHG signal of type I collagen and Gli1^+^ cells were identified by tdTomato (Fig. 3B). Gli1^+^ cells were mainly distributed in the sutures, mesenchyme bone marrow cavity, and periosteum. Notably, Gli1^+^ cells were significantly more abundant in the maxilla than in the zygomatic bone, which was more obvious at 5 and 6 weeks of age. Optical sectioning provided more details regarding the expression patterns of Gli1^+^ cells in the zygomaticomaxillary sutures (Fig. 3B). At 4 weeks of age, Gli1^+^ cells were arranged in an orderly manner at the midline of the zygomaticomaxillary suture and a few were distributed at the OFs and surrounding bone. At 5 weeks of age, the number of Gli1^+^ cells residing in the suture increased in a relatively irregular arrangement. At 6 weeks, Gli1^+^ cells mainly colonized the midline and OFs. Among them, Gli1^+^ cells at the midline were densely distributed, and Gli1^+^ cells at the OFs were arranged in a single row.

### Gli1^+^ cells contribute to osteogenesis induced by distraction force in the zygomaticomaxillary suture

**Fig. 4:**
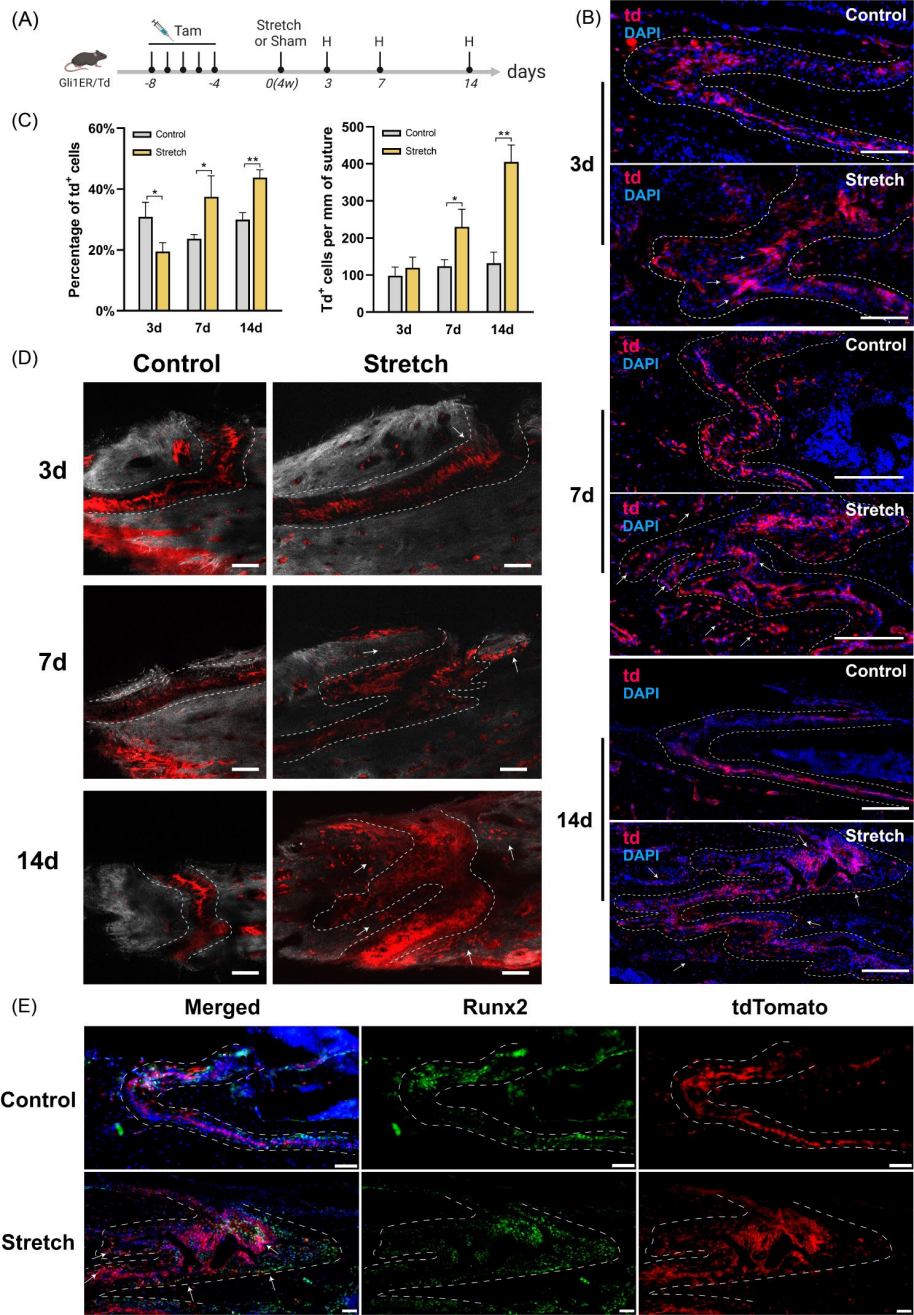
Gli1^+^ cells contribute to osteogenesis induced by distraction force in the zygomaticomaxillary suture (ZMS). (**A**) Experimental design: Gli1ER/Td mice aged 4 weeks were induced with tamoxifen and harvested after 3, 7, and 14 days of stretching. The sham-operated mice were used as control. H: harvested. Tam: tamoxifen. (**B**) The fluorescence images demonstrate the proliferation of Gli1-lineage cells after stretching. DAPI: cell nuclei. td: Gli1-lineage cells. Dotted line: osteogenic fronts (OFs). At 3 days after surgery, arrows indicate elongated cells in Stretch group. At 7 and 14 days after surgery, arrows indicate increased Gli1-lineage cells in OFs and bone areas around sutures in Stretch group. (**C**) Quantitative analysis of the percentage of td + cells and the number of td + cells per unit suture length. *P < 0.05, **P < 0.01. (**D**) Two-photon laser scanning images demonstrate the contribution of Gli1^+^ cells in osteogenesis induced by distraction force. At 3 days after surgery, the arrow indicates elongated Gli1-lineage cells and their connection to the OFs. At 7 and 14 days after surgery, the arrows indicate a large number of Gli1-lineage cells distributed in the bone around the suture. Red: Gli1-lineage cells. Gray: second harmonic signal (SHG) of bone collagen. Dotted line: OFs. Scale bar: 100 μm. (**E**) Immunofluorescence images demonstrate the distribution of Gli1-lineage cells (red) and Runx2^+^ cells (green) after 14 days of stretching. Arrows: Runx2^+^tdTomato^+^ cells. Scale bar: 50 μm. Dotted line: OFs

Gli1ER/Td mice were used to trace the fate of Gli1-lineage cells (Fig. 4A). The fluorescence results showed that many td^+^ cells were present in the OFs and bone areas around the sutures after stretching for 7 and 14 days (Fig. 4B). Quantification of the percentage of td^+^ cells in suture showed a decrease after stretching for 3 days compared to that in controls (19.47 ± 2.86% vs. 30.94 ± 4.76%) and an increase by both 7 and 14 days (Fig. 4C). Based on the overall proliferation of the mesenchymal cells in the sutures, the number of td^+^ cells per unit suture length was counted to accurately reflect the actual change in the number of td^+^ cells. The results showed no significant difference at 3 days and a significant increase at 7 and 14 days (Fig. 4C). This suggests that distraction force promotes the proliferation of Gli1-lineage cells.

The contribution of Gli1^+^ cells and their descendants to osteogenesis was detected using two-photon laser scanning (Fig. 4D). After stretching for 3 days, Gli1-lineage cells distributed at the midline of the suture were elongated and migrated to the OFs. After stretching for 7 days, Gli1-lineage cells were largely expressed at the interlaced OFs, especially within the new finger-like bones and the suture mesenchyme immediately adjacent to them. After 14 days of distraction, more Gli1-lineage cells were distributed in the sutures and adjacent bone. Newly formed bone in the hump-like protrusions at the OFs was filled with Gli1-lineage cells (Fig. 4D). In the absence of distraction forces, Gli1-lineage cells were mainly concentrated at the midline of the suture, with slow accumulation in the OFs and surrounding bone (Control group).

Immunofluorescence staining was performed to detect the expression of Runx2. After stretching for 3 days, Runx2^+^ cells were predominantly distributed at the OFs and partially co-localized with tdTomato (Figure S1). In the Control group, Runx2 was predominantly distributed within the suture mesenchyme on both sides of Gli1-lineage cells, with less distribution at the suture margin (Figure S1). After 7 days, the distribution of Runx2^+^ cells significantly increased in the Stretch group, and tdTomato^+^/Runx2^+^ cells were distributed in the suture mesenchyme and margins of the finger-like new bone (Figure S1). After stretching for 14 days, the distribution of Runx2^+^ cells was more concentrated at the OFs and inside the hump-like protrusion, and mostly co-localized with tdTomato (Fig. 4E).

Based on the fluorescence images at different time points after TSDO, we observed the most significant changes in Gli1^+^ cells at the midline of the ZMS. Morphological alterations occurred on day 3 of stretching, followed by the proliferation and osteogenic differentiation of Gli1^+^ cells under mechanical transduction. Additionally, at different time points after TSDO, there were consistently dense Gli1-lineage cells at the midline of the suture, with limited co-localization with Runx2, suggesting that Gli1^+^ cells at the midline of the suture play a role in both osteogenesis and self-renewal. Gli1^+^ cells at the OFs also responded early to mechanical stimuli, exhibiting osteogenic differentiation on day 3 of stretching, and subsequently promoting the formation of new bone along with Gli1-lineage cells migrating from the midline to the OFs. Gli1^+^ cells in the bone marrow contributed to new bone limitedly, due to the small percentage of Gli1^+^ cells in the bone marrow space, and there is no significant proliferation of Gli1-lineage cells observed here. In conclusion, these data demonstrated the proliferation and osteogenic differentiation of Gli1-lineage cells in response to mechanical stimulation and their important role in the osteogenic process.

### Hedgehog signaling pathway is involved in the osteogenesis of Gli1^+^ cells induced by mechanical force

#### Distraction force up-regulates the expression of Hedgehog signaling pathway

**Fig. 5:**
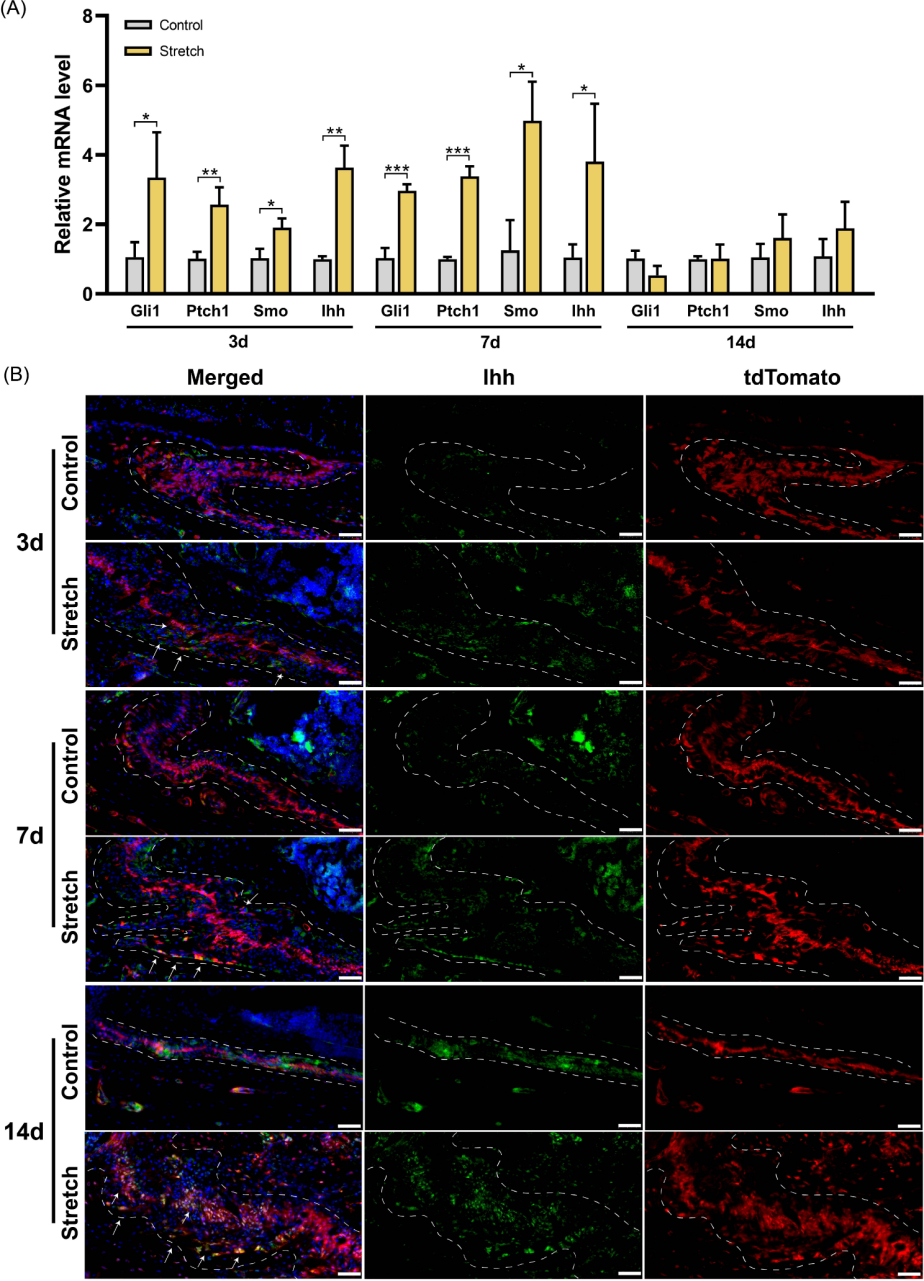
Distraction force up-regulates the expression of Hedgehog signaling pathway. (**A**) RT-qPCR analysis of Hedgehog signaling pathway genes. *P < 0.05, **P < 0.01, ***P < 0.001. (**B**) Immunofluorescence images demonstrate the distribution of Gli1-lineage cells (red) and Ihh^+^ cells (green) after stretching. Arrows indicate Ihh^+^tdTomato^+^ cells. Scale bar: 50 μm. Dotted line: osteogenic fronts (OFs)

Gli1 expression is a reporter for Hh signaling pathway [31]. To investigate whether Hh signaling pathway is involved in the osteogenesis induced by distraction force, we measured the expression levels of genes involved in Hh signaling. The results indicated that the expression levels of Gli1, Ptch1, Smo, and Ihh were significantly up-regulated after stretching for 3 and 7 days (Fig. 5A). By day 14, the expression of Hh signaling was not significantly different between the two groups. Immunofluorescence staining showed that after stretching for 3 days, the number of Ihh^+^ cells in sutures increased (Fig. 5B). After 7 days of distraction, the expression of Ihh was activated more clearly and was mainly distributed in the OFs. After 14 days of distraction, Ihh^+^ cells had the same distribution pattern as Gli1-lineage cells, and most of them co-expressed tdTomato. These data demonstrate that the Hedgehog signaling pathway is activated by mechanical force in Gli1-lineage cells in the zygomaticomaxillary suture, especially at the early stage of distraction.

#### Construction of Hedgehog signaling pathway inhibition TSDO model

**Fig. 6:**
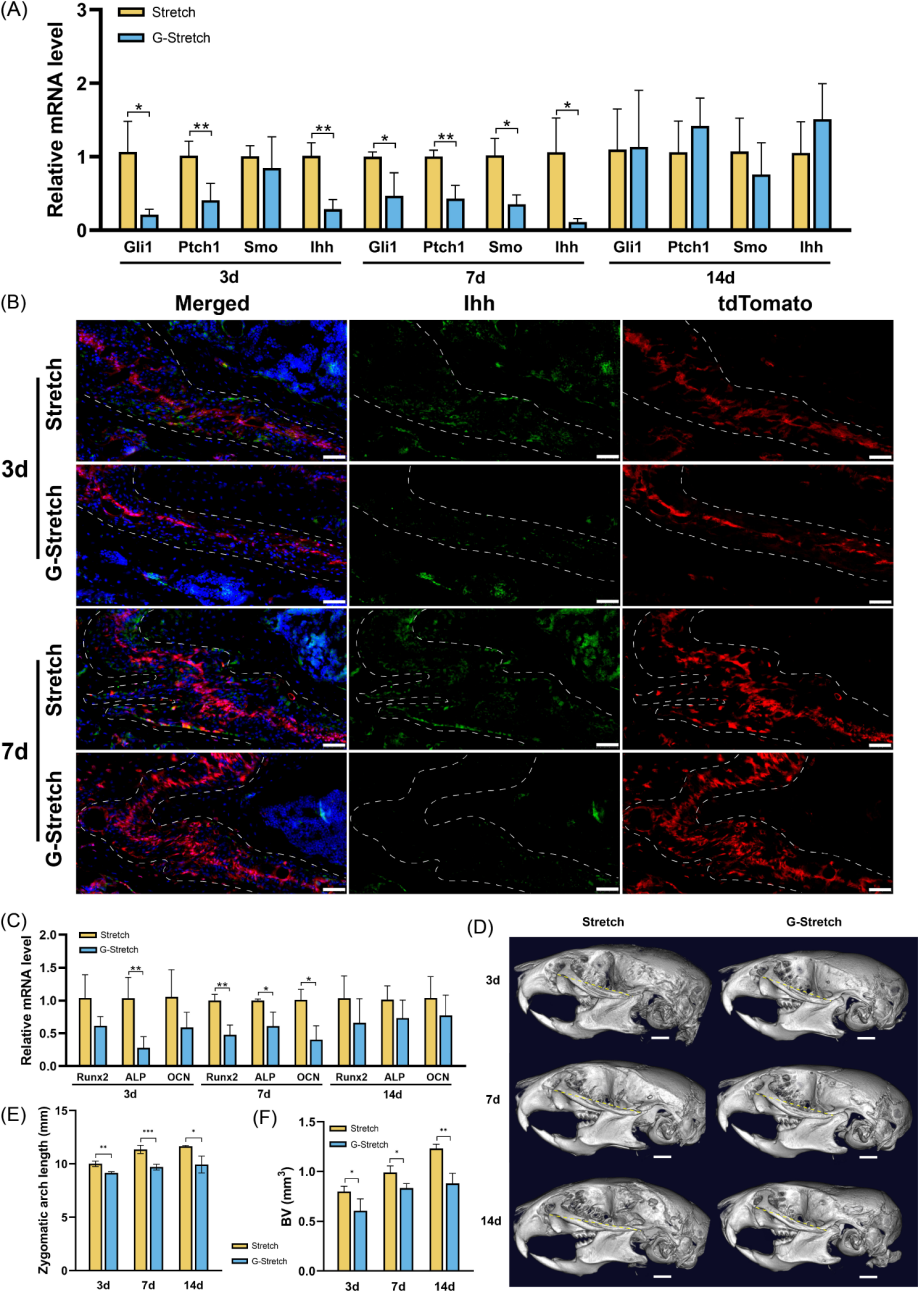
The inhibition of Hedgehog signaling pathway impeded the bone remodeling induced by distraction force. (**A**) RT-qPCR results demonstrate that the inhibition of Gli1 down-regulates the Hedgehog signaling. *P < 0.05, **P < 0.01. (**B**) Immunofluorescence images demonstrate the distribution of Gli1-lineage cells (red) and Ihh^+^ cells (green). Scale bar: 50 μm. Dotted line: osteogenic fronts (OFs). (**C**) RT-qPCR analysis of osteogenesis-related genes of zygomaticomaxillary suture (ZMS) at 3, 7, and 14 days after surgery. *P < 0.05, **P < 0.01. (**D**) Micro-CT scanning of the skull after 3, 7, and 14 days of surgery. Yellow dotted line: length of zygomatic arch. Scale bar: 1 mm. (**E**) Changes in the length of the zygomatic arch. (**F**) Changes in the bone volume (BV) around ZMS. *P < 0.05, **P < 0.01, ***P < 0.001

To further verify whether the Hedgehog signaling pathway participates in regulating SuSCs in zygomaticomaxillary sutures, we treated stretched mice with GANT61 (G-Stretch group) to inhibit the Hedgehog signaling pathway and harvested the tissues at 3, 7, and 14 days after surgery. In the G-Stretch group, Gli1, Ptch1, and Ihh were downregulated on days 3 and 7 compared to that in the Stretch group (Fig. 6A). Smo expression was not significantly different between the Stretch and G-Stretch groups at day 3, but was downregulated at day 7. By day 14, the expression of Hh signaling components did not differ significantly between the two groups (Fig. 6A). Immunofluorescence staining showed that by 3 and 7 days, the Ihh^+^ cells in the suture were significantly decreased in the G-Stretch group (Fig. 6B). By day 14, some Ihh^+^ cells were distributed in the suture and at the OFs, but barely co-localized with tdTomato (Figure S2A). These results indicated that the inhibition of Gli1 suppressed the up-regulation of gene expression in the Hedgehog signaling pathway induced by distraction force, especially in Gli1^+^ cells.

#### The inhibition of Hedgehog signaling pathway impeded the bone remodeling induced by distraction force

HE and Masson staining were performed to observe morphological changes and osteogenesis in the G-Stretch group. Compared to the observations in the Stretch group, the G-Stretch group showed a decrease in the number of cells within the suture, and no newly formed bones were observed at OFs (Figure S2B, C). Analysis of osteogenesis-related gene expression in sutures revealed a decrease in ALP in the G-Stretch group compared to that in the Stretch group 3 days post-surgery (Fig. 6C). At day 7, Alp, Runx2, and OCN were downregulated compared to those in the Stretch group. There was no significant difference between the two groups at 14 days (Fig. 6C). Reconstructed 3D models after MicroCT scanning showed that suture widening in the G-Stretch group was similar to that in the Stretch group in the early stage (Figure S2D); however, the length of the suture and zygomatic arch was shorter in the G-Stretch group (Fig. 6D). Analysis of the zygomatic arch length showed a significant decrease in the G-stretch group compared to that in the Stretch group (Fig. 6E), suggesting that GANT61 inhibited midfacial growth in the sagittal direction promoted by distraction force. The BV on 3, 7, and 14 was significantly lower than that of the Stretch group (Fig. 6F), while there was no significant change in BMD (Figure S2E). These results demonstrate that inhibition of Hedgehog signaling pathway impedes osteogenesis induced by distraction force.

#### The effect of inhibiting the Hedgehog signaling pathway on Gli1^+^ cells during TSDO process

**Fig. 7:**
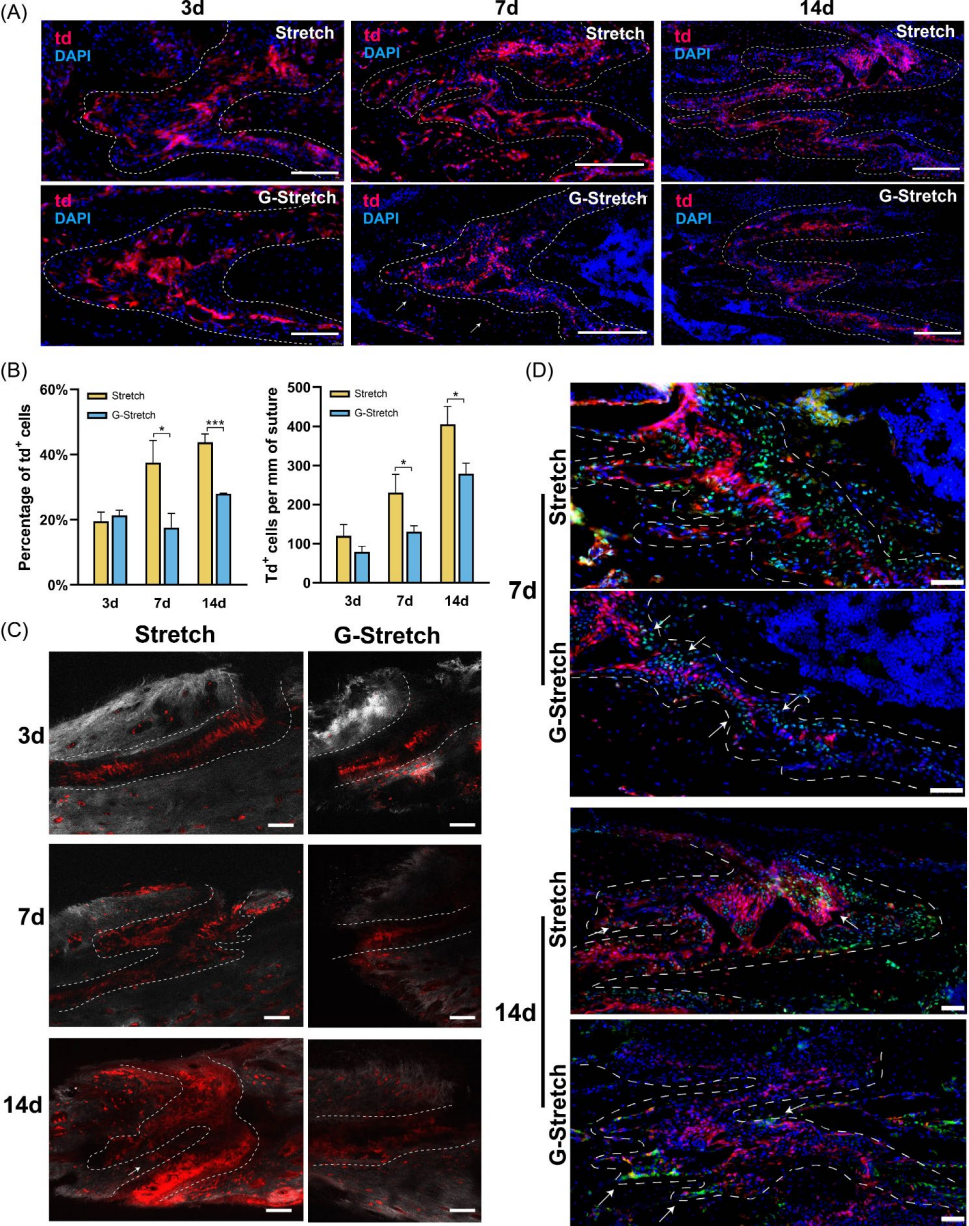
The effect of inhibiting the Hedgehog signaling on Gli1^+^ cells during TSDO process. (**A**) The fluorescence images demonstrate that the proliferation of Gli1-lineage cells was inhibited in the G-Stretch group. DAPI: cell nuclei. td: Gli1-lineage cells. Dotted line: osteogenic fronts (OFs). Arrows indicate the decrease of Gli1-lineage cells in the bone areas around sutures in G-Stretch group. elongated cells in Stretch group. (**B**) Quantitative analysis of the percentage of td + cells and the number of td + cells per unit suture length. *P < 0.05, ***P < 0.001. (**C**) Two-photon laser scanning images demonstrate the reduced Gli1-lineage cells in osteogenesis induced by distraction force in G-Stretch group. Red: Gli1-lineage cells. Gray: second harmonic signal (SHG) of bone collagen. Dotted line: OFs. Scale bar: 100 μm. (**D**) Immunofluorescence images demonstrate the distribution of Gli1-lineage cells (red) and Runx2^+^ cells (green). Dotted line: OFs. Scale bar: 50 μm. At 7 days after surgery, the arrows indicate Runx2^+^ cells not co-located with tdTomato. At 14 days after surgery, the arrows indicate the Runx2^+^ cells at OFs

To further elucidate the effect of inhibition of Hedgehog signaling on Gli1^+^ cells, we observed and analyzed the fluorescence and two-photon images of Gli1ER/Td mice in the G-Stretch group. The results showed at 7 and 14 days postoperatively, a significant reduction in td^+^ cells in the suture was observed in the G-Stretch group compared to the Stretch group (Fig. 7A, B), with no significant aggregation at the OFs and relatively few td^+^ cells in the bone around the suture (Fig. 7C). These results indicated that inhibition of Hedgehog signaling suppressed the proliferation of Gli1-lineage cells induced by distraction force.

In the immunofluorescence images, we observed that the inhibition of Gli1 resulted in a decrease in Runx2^+^/tdTomato^+^ cells, suggesting that the inhibition of Hedgehog signaling decreased the osteogenic differentiation of Gli1^+^ cells (Fig. 7D, Figure S2F). In conclusion, our data demonstrated that GANT61 inhibits the proliferation and osteogenesis of Gli1^+^ cells in response to mechanical force stimulation, further validating the important role of the Hh signaling pathway in this process.

### Gli1^+^ cells are a subset of MSC in zygomaticomaxillary suture

We next investigated whether Gli1^+^ cells in the zygomaticomaxillary suture possessed MSC characteristics. For these experiments, 4-week-old tamoxifen-induced Gli1ER/Td mice were executed to obtain SuSCs. Flow cytometry showed that SuSCs highly expressed MSC markers such as CD90, CD44, and Sca1, but only partially expressed CD29 (19.18%). For negative MSC markers, SuSCs lowly expressed CD31, CD34, and CD117 (Figure S3A). We sorted out purified Gli1^+^ cells by FACS. Slightly different from SuSCs, these cells exhibited a higher CD44 positive rate and lower Sca1 and CD29 positive rates. As for negative markers, Gli1^+^ cells lowly expressed CD31, CD34, and CD117 (Figure S3B). Clonal culture indicated that Gli1^+^ cells possessed the clone-forming ability (Figure S3C). Gli1^+^ cells also exhibit osteogenic, adipogenic, and chondrogenic differentiation abilities (Figure S3C). In conclusion, these data suggested that Gli1^+^ cells express MSC surface markers and possess self-renewal and multilineage differentiation abilities.

### Mechanical force promotes osteogenic differentiation of Gli1^+^ cells via the Hedgehog signaling pathway in vitro

**Fig. 8:**
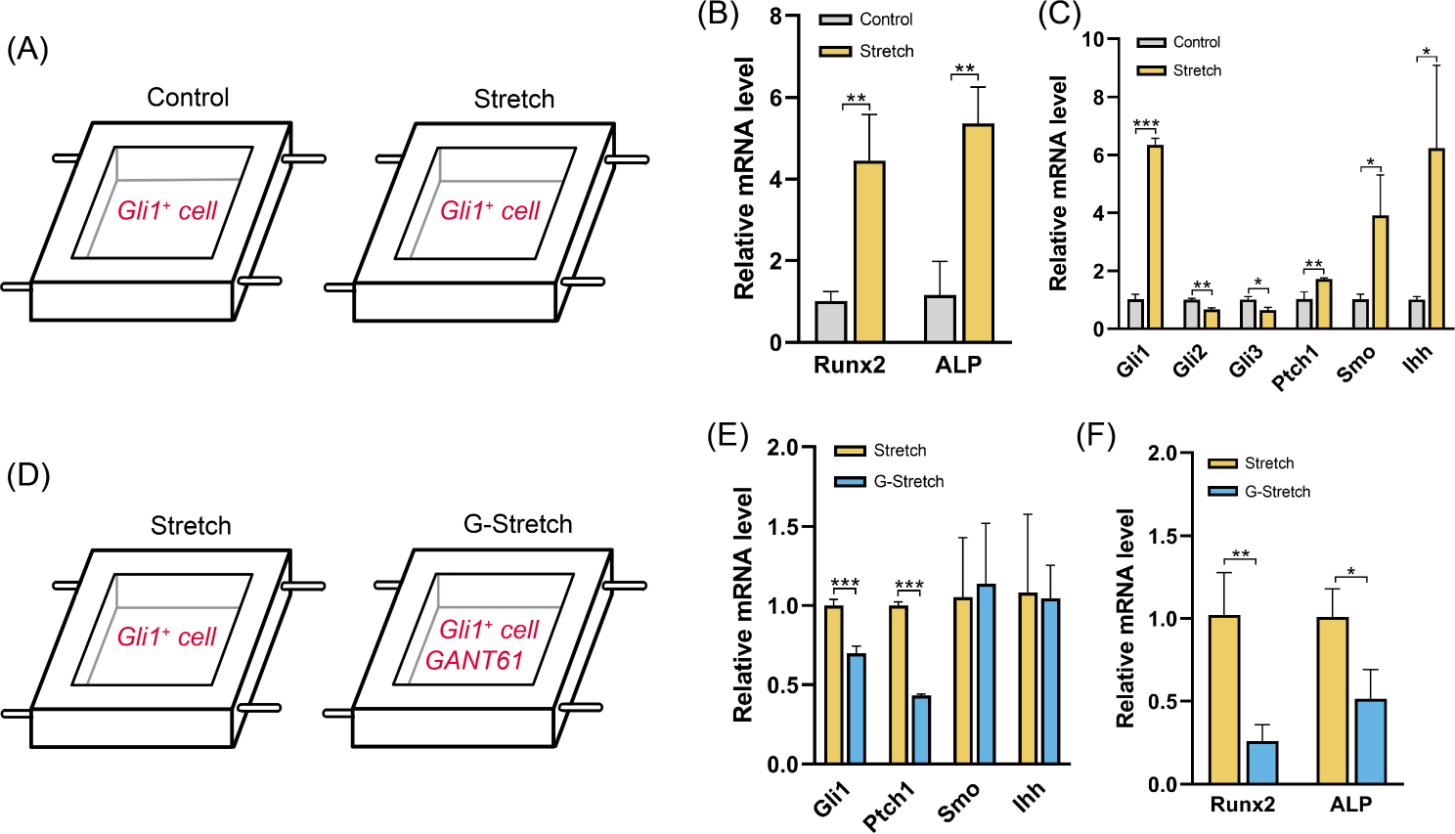
Mechanical force promotes osteogenic differentiation of Gli1^+^ cells through Hedgehog signaling in vitro. (**A**) The schematic diagram of cell stretching in vitro. (**B**) RT-qPCR analysis of osteogenic-related genes. **P < 0.01. (**C**) RT-qPCR analysis of Hedgehog signaling pathway genes. *P < 0.05, **P < 0.01, ***P < 0.001. (**D**) The schematic diagram of the inhibition of Hedgehog signaling during stretching in vitro. (**E**) RT-qPCR analysis of Hedgehog signaling pathway genes. ***P < 0.001. (**F**) RT-qPCR analysis of osteogenic-related genes. *P < 0.05, **P < 0.01

We isolated Gli1^+^ cells and performed in vitro stretching experiments to verify their response to mechanical force stimulation. We inoculated Gli1^+^ cells into cell-stretching chambers according to the schematic diagram (Fig. 8A) and analyzed the gene expression levels of the cells after 6 h per day for 3 days of sine wave mechanical stimulation. The stretching parameters and stretching period in vitro were referenced from the study by Huang et al. [32]. In the pre-experiment, we tested 5%, 8%, and 10% elongation for Gli1^+^ cells and conducted different stretching periods, including 3 and 7 days. We found that extensive elongation and long-term stretching led to cell detachment from the chamber. Consequently, we chose 8% elongation strain and 3 d as the final experimental parameters. The results showed that both Runx2 and ALP expression were significantly upregulated in Gli1^+^ cells under mechanical stimulation, and the expression of Hh signaling components (Gli1, Ptch1, Ihh, and Smo) was also upregulated (Fig. 8B, C). In Gli family (Gli1, Gli2, and Gli3), the transcription factor Gli1 mainly acts as a pure activator. Gli2 and Gli3 are thought to act as both full-length activator forms and truncated repressor forms [33]. We measured the expression of Gli2 and Gli3. The results showed a downregulated expression of both Gli2 and Gli3 (Fig. 8C). This suggests that during mechanical force-induced osteogenesis, Gli1 predominantly plays the role of activating the Hedgehog signaling pathway, whereas Gli2/3 may exist in repressor forms. The mammalian target of rapamycin (mTOR) can facilitate the dissociation of Gli1 from SUFU by phosphorylation, promoting nuclear translocation and enhancing Gli1 activity [34]. We investigated mTOR expression under mechanical force. However, there were no significant differences in the mTOR expression between the Stretch and Control groups (Figure S4). To further verify the involvement of Hh in mechanically induced osteogenic differentiation of Gli1^+^ cells in vitro, we administered GANT61 while stretching cells (G-Stretch) and analyzed gene expression levels (Fig. 8D), which showed that the expression of Runx2 and ALP was significantly downregulated (Fig. 8E). The expression of Gli1 and PTCH1, reporter genes of the Hh signaling pathway, decreased, while the expression of Smo and Ihh did not change significantly (Fig. 8F). Collectively, these results suggest that Gli1^+^ cells respond to mechanical force stimulation with a tendency toward osteogenic differentiation in the absence of niche support provided by sutures and other SuSCs and that Hh signaling is involved in this process.

### Primary cilia in Gli1^+^ cells exhibit Hedgehog-independent mechanosensitivity

**Fig. 9:**
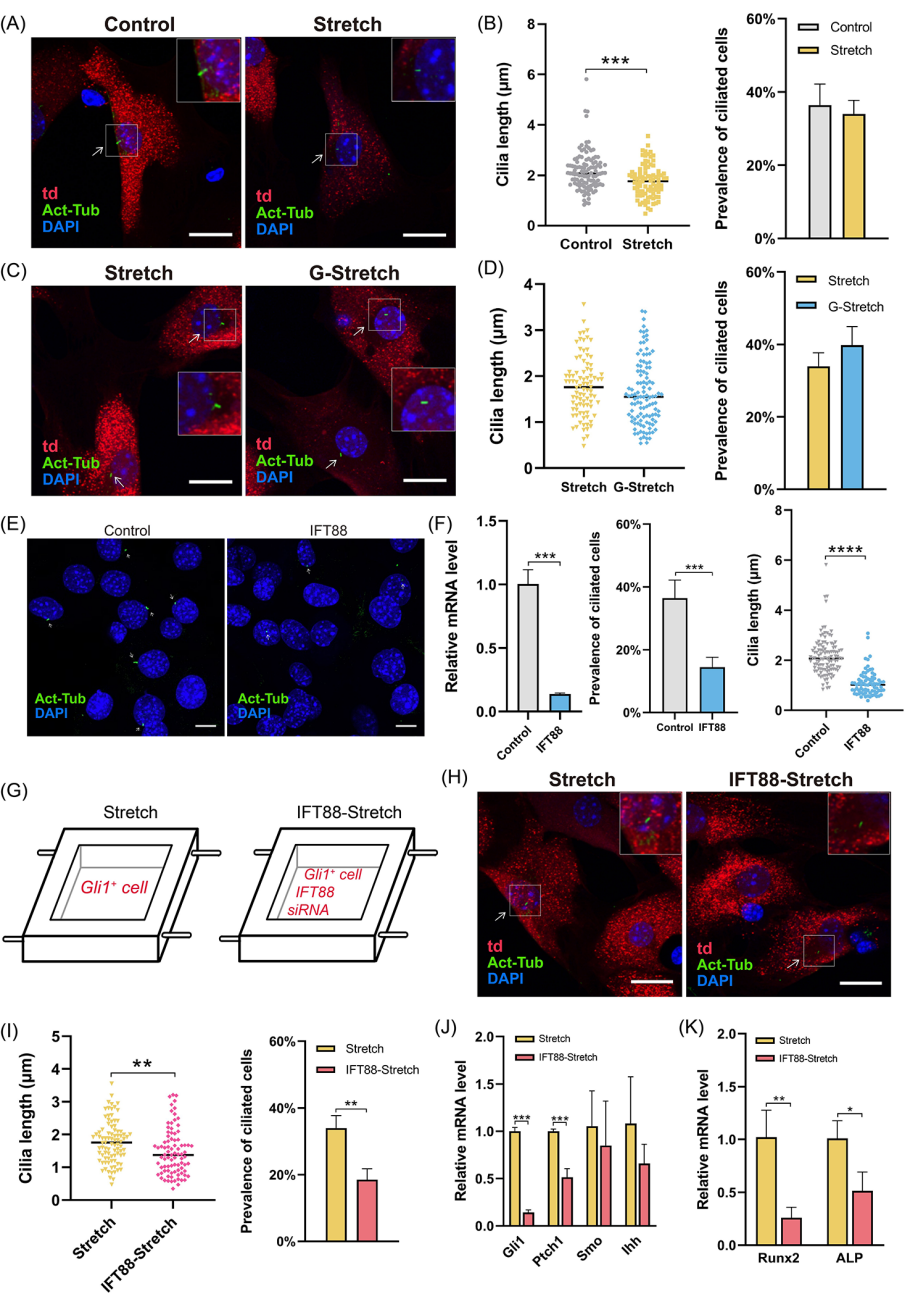
Primary cilia regulate the mechanotransduction in Gli1^+^ cells. (**A**) Immunofluorescence images demonstrate the changes in primary cilia. Regions in boxes are magnified on the right side. td: Gli1^+^ cells. Act-Tub: primary cilia. DAPI: cell nuclei. Scale bar: 20 μm. (**B**) Quantitative analysis of the length of primary cilia and the prevalence of ciliated cells. ***P < 0.001. (**C**) Immunofluorescence images of primary cilia in Stretch group and G-Stretch group. Scale bar: 20 μm. (**D**) Quantitative analysis of the length of primary cilia and the prevalence of ciliated cells. (**E**) Gli1^+^ cells were transfected with siRNA targeting IFT88 to inhibit the primary cilia. Scale bar: 5 μm. (**F**) The mRNA levels of IFT88 decreased. The prevalence of ciliated cells was significantly reduced and the primary cilia length became shorter. ***P < 0.001, ****P < 0.0001. (**G**) The schematic diagram of the inhibition of primary cilia during stretching in vitro. (**H**) Immunofluorescence images of primary cilia in Stretch group and IFT88-Stretch group. Scale bar: 20 μm. (**I**) Quantitative analysis of the length of primary cilia and the prevalence of ciliated cells. **P < 0.01. (**J**) RT-qPCR analysis of Hedgehog signaling pathway genes. ***P < 0.001. (**K**) RT-qPCR analysis of osteogenic-related genes. *P < 0.05, **P < 0.01

To investigate whether primary cilia were mechanosensory in Gli1^+^ cells, we performed acetylated microtubulin (Act-Tub) immunofluorescence staining to analyze the length of primary cilia and the prevalence of ciliated cells after stretching (Fig. 9A, B). The prevalence of ciliated cells in the Control and Stretch groups was 36.4% and 34.0%, respectively, with no significant differences. Under stretched conditions, Gli1^+^ cells expressed a shorter mean cilia length than that in the Control group. These data indicate that the primary cilia in Gli1^+^ cells exhibit mechanosensitivity, as evidenced by the shortened length upon force loading. Subsequently, we explored the relevance of Hh signaling in mechanical force-induced changes of primary cilia using GANT61 (Fig. 9C). The results indicated that the length of primary cilia and the prevalence of ciliated cells were unaffected by Hh inhibition during mechanical force stimulation of Gli1^+^ cells (Fig. 9D). Based on these results, we propose that mechanical force regulates the length of primary cilia via a Hedgehog-independent mechanism.

### Primary cilia are required for the osteogenic differentiation induced by mechanical force in Gli1^+^ cells

To further investigate the role of primary cilia in mechanotransduction, Gli1^+^ cells were transfected with siRNA targeting IFT88 (IFT88-Stretch group). Figure 9E confirmed the successful inhibition of primary cilia: after transfection with siRNA targeting IFT88 (IFT88 group), the prevalence of ciliated cells was significantly reduced, the primary cilia length became shorter, and the mRNA levels of IFT88 significantly decreased (Fig. 9F). Gli1^+^ cells transfected with siRNA targeting IFT88 were subjected to stretching for 6 h per day for 3 days, changes in primary cilia were measured, and the expression of Hh signaling and osteogenesis-related genes was analyzed (Fig. 9G, H). The results showed that primary cilia length and incidence were significantly lower in the IFT88-Stretch group than in the Stretch group (Fig. 9I). The expression levels of Gli1, Ptch1, Runx2, and ALP also decreased (Fig. 9J, K). This suggests that inhibition of primary cilia downregulates mechanical force-induced Hh signaling and osteogenesis-related gene expression. Interestingly, for a small number of ciliated Gli1^+^ cells transfected with siRNA, the primary cilia length of these cells increased in response to mechanical force stimulation, in contrast to the trend observed in untransfected Gli1^+^ cells (Figure S5). This indicates that, in the case of inhibition and shortening of primary cilia, mechanical force promotes primary cilia length recovery. In conclusion, these results demonstrated that in Gli1^+^ cells, primary cilia exhibit adaptive changes in length as mechanosensors. Primary cilia regulate Hh signaling during mechanotransduction and are required for osteogenic differentiation induced by mechanical force in Gli1^+^ cells.

## Discussion

TSDO can provide distraction force and promote the three-dimensional growth of midfacial bones [35]. However, the intricate biological mechanisms governing the osteogenic processes within TSDO remain inadequately elucidated, consequently impeding the refinement of clinical therapeutic strategies. Gli1^+^ SuSCs have been demonstrated to contribute to new bone formation during cranial bone growth and injury repair [10, 14]. However, research on the distribution and characteristics of Gli1^+^ cells in facial sutures remains limited. This study, for the first time, confirmed the osteogenic potential of Gli1^+^ cells in zygomaticomaxillary sutures in response to distraction force and elucidated the mechanobiological mechanism. Specifically, the force applied at the zygomaticomaxillary suture regulates the proliferation and osteogenic differentiation of Gli1^+^ cells through the Hedgehog signaling pathway, thereby facilitating bone remodeling. In vitro experiments validated these findings and demonstrated that mechanotransduction is mediated by primary cilia.

Extensive studies have demonstrated the pivotal role of Gli1^+^ cells in the growth, development, and injury repair of craniofacial bones, as well as long bones [14, 36–38]. Zhao et al. established Gli1^+^ cells as the primary population of SuSCs and described the distribution pattern of Gli1^+^ cells in craniofacial bones [14]. However, there is a lack of comprehensive research concerning the distribution of Gli1^+^ cells in the zygomaticomaxillary suture and zygomatic arch, as well as how this distribution may change during growth. Our study revealed that Gli1^+^ cells in growing mice are predominantly distributed at the midline of the zygomaticomaxillary suture, within the bone marrow cavity, and in the periosteum. In mice aged 5 to 6 weeks, there were significantly more Gli1^+^ cells in the maxilla than in the zygomatic bones. Skeletal structures serve as attachment sites for muscles that exert mechanical loads on bones [39]. In mice, the masseter muscle is attached to the inferior margin of the zygomatic arch, primarily to the maxilla. Bone tissue adapts its density and structure in response to changes in the mechanical loads [39, 40]. Therefore, mechanical stimulation of masticatory force at sites where the maxillary periosteum interfaces with muscle attachments may lead to the observed distribution pattern of Gli1^+^ cells.

Subsequently, we investigated the osteogenesis of Gli1^+^ cells under distraction force. Indeed, Gli1^+^ cells in various tissues have been established as mechanosensitive and are involved in force-mediated bone remodeling processes [32, 41, 42]. Liu. first identified Gli1^+^ cells in the periodontal ligament (PDL) and demonstrated that Gli1^+^ cells in the PDL are force-responsive, directly reacting to orthodontic forces and mediating bone remodeling [42]. Relevant research has also been conducted on the mechanotransduction of Gli1^+^ cells in cranial sutures. One study reported that Gli1^+^ cells contribute to osteogenesis upon sagittal suture expansion, and that Wnt signaling is crucial to this process [41]. Although the mechanosensitivity of Gli1^+^ cells has been confirmed, it remains largely unknown whether Gli1^+^ cells in the ZMS respond to mechanical force stimulation during TSDO. In this study, we observed that during the early stages of distraction (3 d), Gli1^+^ cells underwent elongation but did not exhibit significant proliferation. However, in the sagittal suture expansion model, Gli1-lineage cells exhibited substantial proliferation as early as 1 d after distraction, suggesting that Gli1^+^ cells in the facial sutures may respond to mechanical forces with delayed timing compared with cranial sutures [41]. As mechanical stimulation continued, Gli1^+^ cells exhibited significant proliferation at 7- and 14-days post-surgery. Runx2 is a transcription factor that serves as a marker of osteoblast/osteoprogenitor cells. By leveraging lineage-tracing techniques, we observed a co-localization relationship between Gli1-lineage cells and Runx2^+^ cells in tissue sections, confirming the osteogenic differentiation of Gli1^+^ cells within the ZMS. In the Stretch group, the trend in Runx2 transcription seemed inconsistent with the number of Runx2^+^ cells. Similarly, as reported by Huang et al., although there was no significant difference in Runx2 mRNA levels during rapid maxilla expansion, immunofluorescence staining results indicated an increase in Runx2 expression [32]. We speculate that this may be related to post-transcriptional modifications of Runx2, such as acetylation of Runx2 mRNA [43]. Gli1-lineage cells co-localized with Runx2 were concentrated at the OFs and surrounding bone, indicating their osteogenic differentiation. Moreover, osteogenesis of Gli1^+^ cells contributed to bone remodeling, resulting in zygomatic arch elongation.

As widely recognized, Gli1 is a transcription factor in the Hh signaling pathway, one of the most critical signaling pathways associated with craniofacial development and a significant regulatory pathway in skeletal development [19, 44]. Numerous in vitro studies have demonstrated that Hh signaling plays a role in regulating the lineage differentiation fate of skeletal stem/progenitor cells [45–48]. There is also evidence indicating the involvement of Hh signaling in the regulation of osteogenesis in Gli1^+^ cells. In the growth plates of long bones, inhibition of the Hh signaling pathway hinders the proliferation and osteogenic differentiation of Gli1^+^ cells, resulting in decreased bone mass [37]. In cranial sutures, conditional Bmpr1a knockout in Gli1^+^ cells leads to downregulation of Hh signaling pathway and enhances osteogenic differentiation in Gli1^+^ cells [49]. Overall, the current research primarily focuses on the role of Hh signaling in growth and development, and the regulatory effects of Hh signaling on Gli1^+^ cells exhibit significant heterogeneity across different tissues. There is also controversy regarding the Hh ligands regulate osteogenesis. The role of Ihh in endochondral ossification is well established [50]. However, there is also evidence suggesting the crucial role for Ihh in the intramembranous ossification of craniofacial bones [51, 52]. Our study, for the first time, confirmed that distraction force can upregulate the Hh signaling components in the zygomaticomaxillary suture. Activation of the pathway appears in the early stages of distraction and persists for up to 7 days. During this process, Ihh ligand expression increases in the zygomaticomaxillary suture and participates in the regulation of Gli1-lineage cells. Combined with the bone remodeling process, this suggests that distraction forces exert mechanical stimulation on Gli1^+^ cells in the early stage, promoting mechanotransduction, activating the downstream Hh signaling pathway cascade, and directing Gli1^+^ cells towards osteogenic differentiation. There are negative feedback regulatory mechanisms in the Hedgehog pathway. Gli1 transcriptional activation upregulates the expression of the target gene Ptch1 and inhibits the Hh signaling pathway through a negative feedback mechanism [53, 54]. Based on this regulatory mechanism, we believe that the activity of the Hh pathway decreases in the later stage of TSDO (14 d). In addition, the bone remodeling process has been basically completed at 14 days, and the mechanical stimulation of the traction stent to the suture niche decreased, resulting in a decrease in the activity of the Hh pathway with a decrease in the expression of osteogenic-related genes. Inhibition of Hh signaling resulted in a reduction in the number of Gli1-lineage cells in the zygomaticomaxillary suture and impeded the bone remodeling process, confirming the regulatory role of Hh signaling in Gli1^+^ cells. This underscores the critical importance of the Hh signaling pathway in force-mediated zygomaticomaxillary suture bone remodeling.

The microenvironment of sutures includes various cell types such as fibroblasts [55], osteoblasts/osteoprogenitor cells [56], SuSCs [57], vascular endothelial cells [58], and immune cells [59]. Among them, SuSCs encompass distinct cellular subsets and exhibit crosstalk between these different cell populations. However, elucidating the biological mechanisms of specific cell types in this complex environment is challenging. Therefore, we isolated and cultured SuSCs from the ZMS and obtained purified Gli1^+^ cells by FACS, validating their typical MSC characteristics. We established cell stretching model and subjected the purified Gli1^+^ cells to mechanical stretching for the first time in vitro, thereby avoiding the influence of other cell types. Our results confirm that Gli1^+^ cells from the zygomaticomaxillary suture possess the ability to respond to mechanical stimulation and further differentiate towards the osteogenic lineage both in vivo and in vitro. Furthermore, these findings underscore the significant role of the Hh signaling pathway in mediating mechanotransduction in Gli1^+^ cells. Interestingly, in the in vivo TSDO, inhibition of the Hh signaling resulted in a decrease in Ihh ligand expression (G-Stretch vs. Stretch), whereas in vitro experiments, inhibition of the Hh signaling did not show significant changes in Ihh ligand expression (G-Stretch vs. Stretch). Based on the above results, we speculate that mechanical force promotes the upregulation of Ihh in vitro, activating the canonical Hh pathway in Gli1^+^ cells through secretion/paracrine effects. Therefore, GANT61 does not affect Ihh expression. In in vivo experiments, mechanical forces may activate the Hh signaling pathway partly through non-canonical pathways, and Ihh acts as a positive feedback regulator to increase expression. However, it is important to note the differences between in vivo and in vitro environments. In in vivo experiments, the W-type distraction devices apply forces to the zygomatic and maxillary bones, transmitting forces to Gli1^+^ cells in the ZMS through tissue interactions. In vitro stretching experiments, mechanical force directly stimulates Gli1^+^ cells. Thus, confirming whether these stretching parameters match those of the in vivo experiments is challenging.

Research on the mechanotransduction of MSCs is an ever-evolving field [40]. Currently, the proposed mechanosensors include mechanosensitive ion channels, integrins, connexins, lipid rafts, and primary cilia [60, 61]. Regarding the specific mechanism of mechanosensitivity in primary cilia, it is widely believed that under the influence of fluid shear stress, primary cilia undergo bending and shortening, leading to calcium ion influx and cell-cell interactions [62]. The assembly and maintenance of primary cilia requires intraflagellar transport (IFT) protein. The IFT protein complex moves from the base to the tip of primary cilia under the power provided by Kinesin-2, regulating changes in primary cilia length [63]. Modulation of intracellular Ca2^+^ controls cilium length in part by regulating IFT particle transport velocity and possibly material anterograde flux [64]. Some studies have indicated that primary cilia in human mesenchymal stem cells (hMSCs) exhibit mechanosensitivity and mediate osteogenesis [30]. However, mechanosensitivity of primary cilia in SuSCs has not yet been verified. In this study, we subjected Gli1^+^ cells to uniaxial cyclic tensile strain and observed a reduction in cilia length; however, no significant changes in curvature or incidence were observed. Changes in ciliary length reflect a negative feedback mechanism, in which shorter primary cilia have a reduced lever arm length for mechanosensation, resulting in reduced sensitivity to force stimuli [30]. Longer cilia are more likely to exhibit bending or deflection [65]. In this study, the primary cilia length of Gli1^+^ cells was measured at 2.17 ± 0.77 μm, suggesting that their length may not be sufficient to support ciliary bending or deflection in response to mechanical forces. In summary, our findings confirmed for the first time the mechanosensitivity of primary cilia in Gli1^+^ cells of the zygomaticomaxillary suture. These cilia shorten in response to mechanical force.

Primary cilia are the major organelles that coordinate mechanosignaling for Hh signaling pathway activation [66]. We examined the relationship between Hh signaling and changes in primary cilia induced by mechanical force. These results confirmed that inhibiting Hh signaling during mechanical stimulation of Gli1^+^ cells had no impact on the length or occurrence rate of primary cilia. Based on these findings, we propose that mechanical force regulates primary cilia length via a mechanism independent of Hh signaling. In other words, the expression of the Hedgehog signaling does not affect the mechanosensitivity of primary cilia in Gli1^+^ cells. In Gli1^+^ cell stretching experiments with inhibited primary cilia, the Hh signaling pathway was downregulated, and osteogenic differentiation of Gli1^+^ cells was inhibited. This confirms that during mechanical force stimulation of Gli1^+^ cells, primary cilia mediate downstream Hh signaling activation, thereby promoting osteogenic differentiation. It is worth noting that we observed an adaptive regulatory mechanism in the primary cilia. Previous research has reported “stress-deprivation " of primary cilia, where mechanical forces and increased cellular cytoskeletal tension lead to ciliary shortening [67]. However, in our inhibited primary cilia model, we compared the stretched group with the control group and found that ciliary length increased after stretching. This suggests that when primary cilia are inhibited and shortened, mechanical forces promote their length recovery. In summary, these results indicate that primary cilia, which act as mechanosensors in Gli1^+^ cells, exhibit adaptive changes in length. Primary cilia regulate Hh signaling in mechanotransduction, which is essential for force-induced osteogenic differentiation of Gli1^+^ cells.

However, this study has some limitations. First, the exploration of force-induced morphological changes in primary cilia is relatively simple, and there is a lack of in-depth exploration of the mechanisms of regulating Hh signaling. Furthermore, we did not design different stretching parameters or different stretching periods to determine the optimal treatment method, which requires further investigation. We will consider further exploring the optimal distraction parameters and the Hh activation peak time by increasing time points, shortening time intervals, and setting gradient changes in distraction force and periods.

## Conclusions

Based on our findings, we conclude that primary cilia of Gli1^+^ cells sense distraction force stimuli, mediate Hedgehog signaling activation, and promote the proliferation and osteogenic differentiation of Gli1^+^ cells in the zygomaticomaxillary suture.

### Electronic supplementary material

Below is the link to the electronic supplementary material.

Supplementary Material 1

Supplementary Material 2

Supplementary Material 3

Supplementary Material 4

Supplementary Material 5

Supplementary Material 6

Supplementary Material 7

## Data Availability

The datasets used and/or analysed during the current study are available from the corresponding author on reasonable request.

## Abbreviations

TSDO: Trans-sutural distraction osteogenesis
MH: Midfacial hypoplasia
ZMS: Zygomaticomaxillary suture
SuSCs: Suture mesenchymal stem cells
OFs: Osteogenic fronts
Hh: Hedgehog
Dhh: Desert hedgehog
Ihh: Indian hedgehog
Shh: Sonic hedgehog
Ptch1: Patched-1
Smo: Smoothened
BV: Bone volume
BMD: Bone mineral density
SHG: Second harmonic generation
mTOR: Mammalian target of rapamycin
