# Supplementary figures and images for: Distraction force promotes the osteogenic differentiation of Gli1+ cells in facial sutures via primary cilia-mediated Hedgehog signaling pathway
Source: Stem Cell Res Ther. 2024 Jul 6;15:198. doi: 10.1186/s13287-024-03811-3 (PMC11227703; doi:10.1186/s13287-024-03811-3)

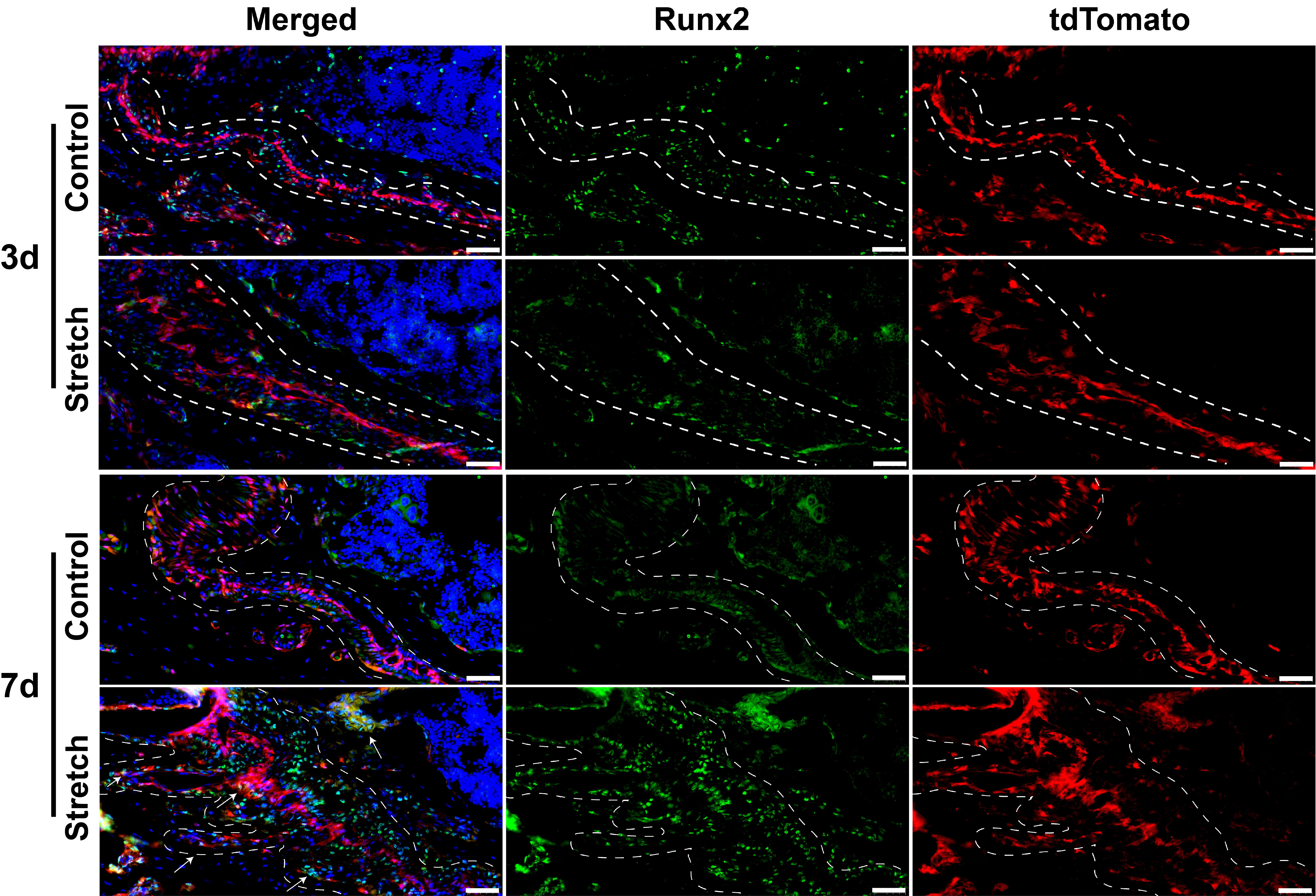

Supplement: Supplementary file 1 — Supplementary Material 1 [file 13287_2024_3811_MOESM1_ESM.jpg]

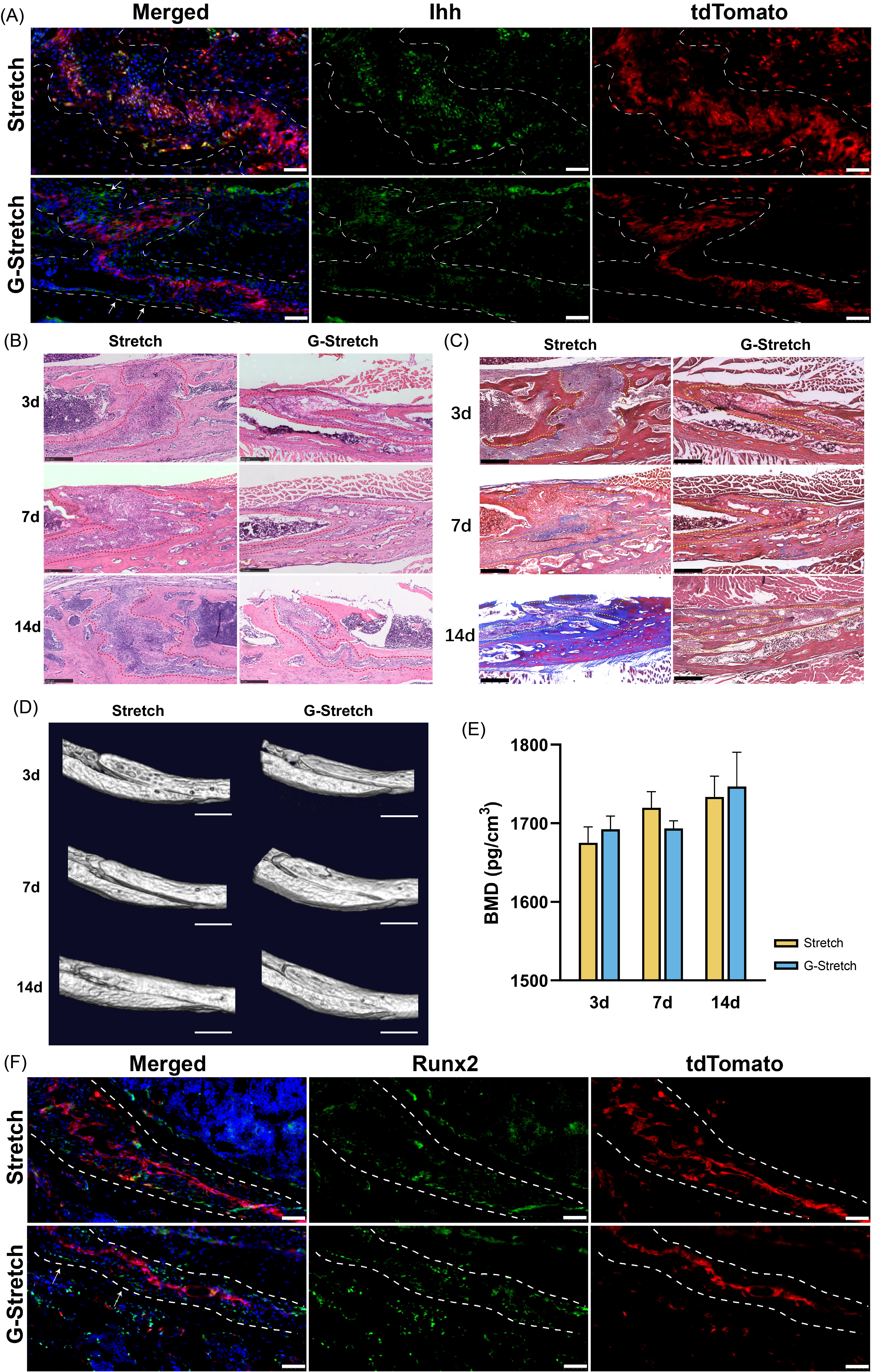

Supplement: Supplementary file 2 — Supplementary Material 2 [file 13287_2024_3811_MOESM2_ESM.jpg]

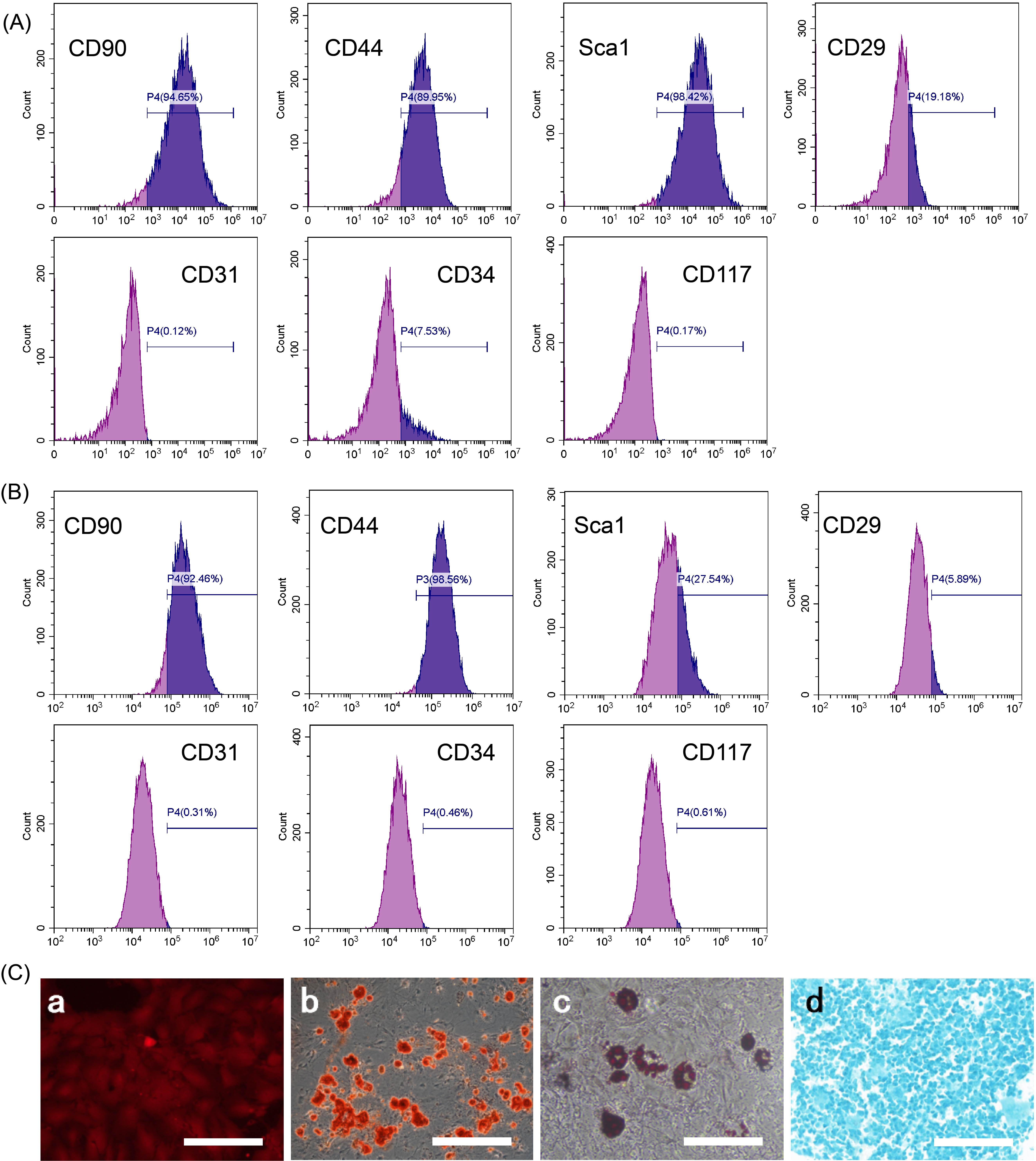

Supplement: Supplementary file 3 — Supplementary Material 3 [file 13287_2024_3811_MOESM3_ESM.jpg]

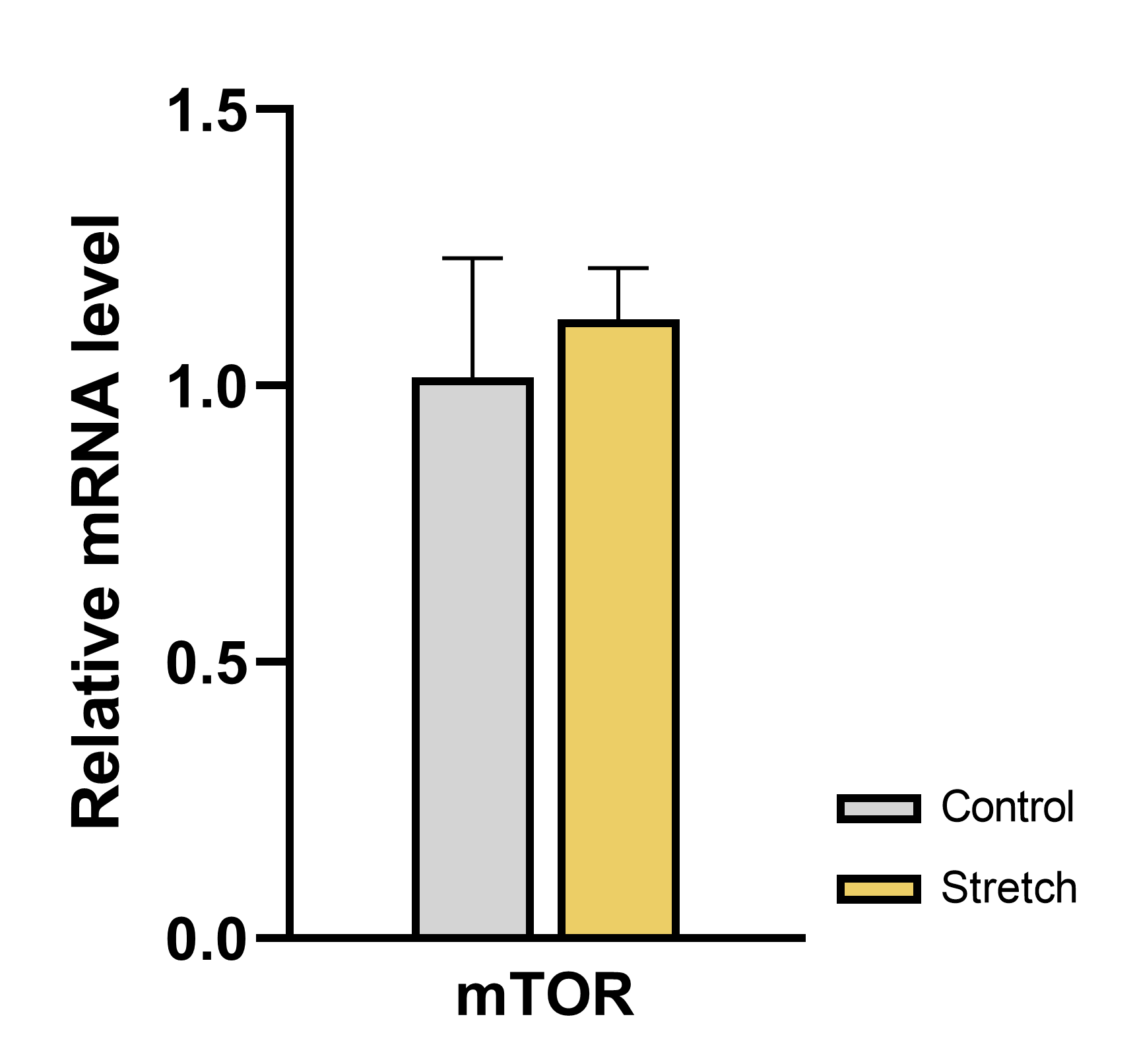

Supplement: Supplementary file 6 — Supplementary Material 6 [file 13287_2024_3811_MOESM6_ESM.tif]

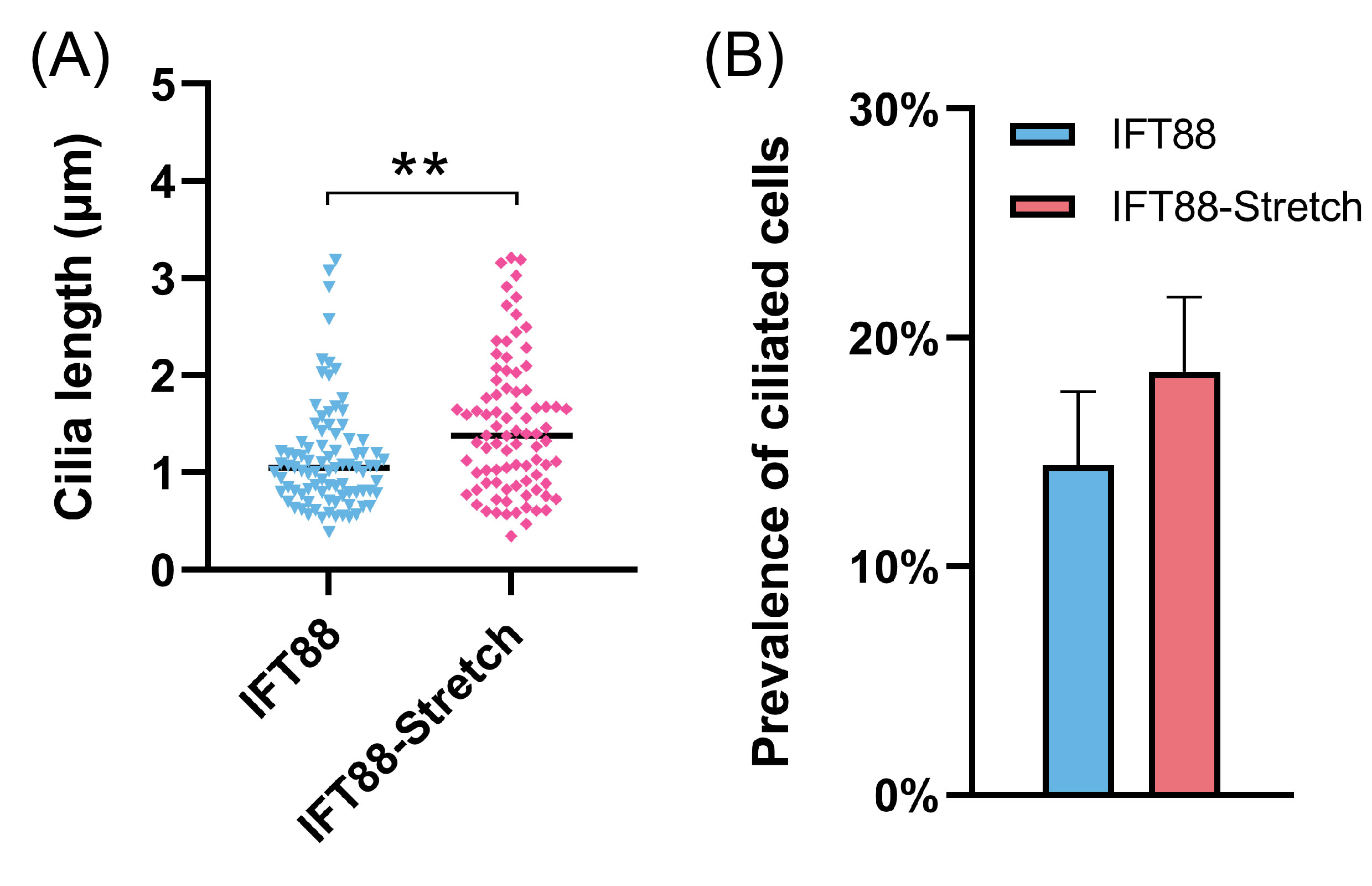

Supplement: Supplementary file 7 — Supplementary Material 7 [file 13287_2024_3811_MOESM7_ESM.jpg]
