# Supplementary material for: Distraction force promotes the osteogenic differentiation of Gli1+ cells in facial sutures via primary cilia-mediated Hedgehog signaling pathway
Source: Stem Cell Res Ther. 2024 Jul 6;15:198. doi: 10.1186/s13287-024-03811-3 (PMC11227703; doi:10.1186/s13287-024-03811-3)
Supplement: Supplementary file 4 — Supplementary Material 4 [file 13287_2024_3811_MOESM4_ESM.docx]

**Supplementary Figure Legends**

Figure S1: Immunofluorescence images demonstrate the distribution of Gli1-lineage cells (red) and Runx2^+^ cells (green) after 3 and 7 days of stretching. Arrows: Runx2^+^tdTomato^+^ cells in osteogenic fronts and bone areas around sutures. Scale bar: 50 µm. Dotted line: OFs.

Figure S2. The inhibition of Hedgehog signaling pathway impeded the force-induced osteogenesis of Gli1^+^ cells. (A) Immunofluorescence images demonstrate the distribution of Gli1-lineage cells (red) and Ihh^+^ cells (green) at 14 days after surgery. Scale bar: 50 µm. Dotted line: osteogenic fronts (OFs). Arrows: Ihh^+^ cells at the OFs barely co-localize with tdTomato. (B-C) Histological changes of ZMS after stretching for 3, 7, and 14 days (HE staining and Masson staining). Dotted line: OFs. Scale bar: 250 µm. (D) Micro-CT scanning of ZMS after 3, 7, and 14  days of surgery. Yellow dotted line: length of zygomatic arch. Scale bar: 1 mm. (E) Changes in the bone mineral density (BMD) of the bone around ZMS. (F) Immunofluorescence images of Gli1-lineage cells (red) and Runx2^+^ cells (green) after 3 days of surgery. Scale bar: 50 µm. Dotted line: OFs. Arrows: Runx2^+^ cells at the OFs barely co-localize with tdTomato.

Figure S3. Gli1^+^ cells possess mesenchymal stem cells (MSC) characteristics. Flow cytometry analysis of the surface markers of SuSCs (A) and Gli1^+^ cells (B). (C) a: Gli1^+^ cells exhibit clone-forming ability. b-d: Gli1^+^ cells exhibit osteogenic, adipogenic, and chondrogenic differentiation abilities.

Figure S4. RT-qPCR analysis of mTOR.

Figure S5. The changes of inhibited cilia after stretching. (A) Quantitative analysis of the length of primary cilia. **P<0.01. (B) Quantitative analysis of the prevalence of ciliated cells.
