# Supplementary material for: Distraction force promotes the osteogenic differentiation of Gli1+ cells in facial sutures via primary cilia-mediated Hedgehog signaling pathway
Source: Stem Cell Res Ther. 2024 Jul 6;15:198. doi: 10.1186/s13287-024-03811-3 (PMC11227703; doi:10.1186/s13287-024-03811-3)
Supplement: Supplementary file 5 — Supplementary Material 5 [file 13287_2024_3811_MOESM5_ESM.docx]

| Gene | Forward | Reverse |
| --- | --- | --- |
| GAPDH | AGGTCGGTGTGAACGGATTTG | GGGGTCGTTGATGGCAACA |
| Runx2 | GACTGTGGTTACCGTCATGGC | ACTTGGTTTTTCATAACAGCGGA |
| ALP | CCAACTCTTTTGTGCCAGAGA | GGCTACATTGGTGTTGAGCTTTT |
| OCN | AAGCAGGAGGGCAATAAGGT | ACTTGCAGGGCAGAGAGAGA |
| Gli1 | CCAAGCCAACTTTATGTCAGGG | AGCCCGCTTCTTTGTTAATTTG |
| Gli2 | ACCCCTGATCCAGCCTTCA | GTTGGCATCATTTAGACAGTTGC |
| Gli3 | GAAGAAACGCAATCACTATGCAG | GTCCCACGGTAAGGGAGAGA |
| Smo | GTGCTGTCTACATGCCCAAGT | GCAACGCAGAAAGTCAGGC |
| Ptch1 | GCCTTCGCTGTGGGATTAAAG | CTTCTCCTATCTTCTGACGGGT |
| Ihh | CTCTTGCCTACAAGCAGTTCA | CCGTGTTCTCCTCGTCCTT |
| mTOR | CAGTTCGCCAGTGGACTGAAG | GCTGGTCATAGAAGCGAGTAGAC |
| IFT88 | TGGCCAACGACCTGGAGATTAACA | ATAGCTGCTGGCTTGGGCAAATTC |

Table SI. List of primers used for RT-qPCR for mRNAs.
